# Assessment of cattle traceability using electronic identification and digital movement reporting in simulated United States cattle movement networks

**DOI:** 10.3389/fvets.2026.1972037

**Published:** 2026-10-27

**Authors:** MaRyka R. Smith, Michael W. Sanderson, Christy J. Hanthorn

**Affiliations:** College of Veterinary Medicine, Diagnostic Medicine and Pathobiology, Center for Outcomes Research and Epidemiology, Kansas State University, Manhattan, KS, United States

**Keywords:** cattle, electronic identification, livestock movement network modeling, network, traceability

## Abstract

**Introduction:**

In a disease outbreak, animal health officials in the United States (US) must identify the diseased and at-risk animals and implement control measures. For rapidly-spreading diseases, electronic identification (EID) of animals and digital reporting (DR) of animal movements are likely needed for rapid, effective tracing and control measure implementation. Currently, the US does not require all cattle movements to be reported and only requires official EID of dairy cattle and some classes of beef cattle. This project created a simulated national cattle movement network to assess the traceability of farms with various levels of EID and DR.

**Methods:**

A 30-day, simulated US cattle movement network was generated. In the network, farms, markets, processors, and other premises are nodes and movements between premises are edges. A random sample of 100 cow-calf farms, 100 markets, and 100 feedlots were selected from the full network to be “farms of interest” (FOIs) to assess traceability to their contact farms. To generate traceable networks, EID status was assigned to source nodes and DR status assigned to destination nodes using a binomial process. The EID and DR probabilities began with a baseline level that represented the estimated current usage in US cattle, and improved across increasing EID and DR probabilities to the highest level wherein 75% of all beef cattle farms tagged outgoing movements and 90% reported incoming movements. A movement was traceable and included in the traceable network when the source tagged and the destination reported.

**Results and discussion:**

In the baseline scenario, there were zero traceable incoming contacts across all three farm types tested. As EID and DR increased, the traceability increased. With the highest level of EID and DR, the average percent of contacts traceable was 83.56% for cow-calf FOIs, 86.5% for market FOI total contacts, and 75.63% for feedlot FOIs. These results support increasing EID tag use and DR to enable rapid, effective tracing and provide implementation targets to improve traceability. The current EID and DR adoption is likely insufficient for effective electronic tracing and higher usage is needed to facilitate rapid tracing.

## 1 Introduction

Animal disease traceability (ADT) has been defined by the United States Department of Agriculture (USDA) as, “knowing where diseased and at-risk animals are, where they have been, and when they were there” (1). An efficient and accurate tracing system improves the deployment of disease control resources which can impact the outcomes of a disease outbreak (1). Rapid tracing can be facilitated by electronic identification (EID) of animals and digital reporting (DR) their movements, including the source and destination location, to a searchable electronic database. Currently in the United States (US), there is low adoption of digital identification of beef cattle. Additionally, the movement records accessible to animal health officials for beef cattle are often incomplete. This leads to ineffective electronic traces and variable, often extended, time periods required to manually complete trace events (2).

The USDA ADT program was implemented over 10 years ago and has resulted in substantial gains in the speed and accuracy of tracing cattle covered under the rule (cattle >18 months of age and sexually intact, dairy cattle of any age, and all cattle for show or exhibition) (2). More recently, the 2024 ADT final rule now requires that covered animals have official identification tags that are both visually and electronically readable (3). However, beef calves less than 18 months of age are exempt from the rule requiring official identification, and therefore are less digitally traceable. Further, in many states, movements that do not cross state lines and movements directly to slaughter are exempt from reporting. These identification and reporting exemptions leave a potentially large number of cattle movements digitally untraceable. Tracing the movement of animals that are digitally untraceable requires labor and time intensive manual methods.

In addition to the federal ADT rules, the US beef industry has shown increasing desire to improve cattle traceability in recent years. The 2016–2020 Beef Industry Long Range Plan listed improving traceability as a priority and World Perspectives, Inc., completed a comprehensive study on the feasibility of cattle traceability (4). The group found that despite being a “globally-recognized leader” in beef production the US was lacking a robust traceability system for the domestic beef industry and estimated that 45-75% of producers adopting traceability technology could result in 35–90% of US beef being traceable (4). The reported stated this range was “Nationally significant and economically efficient.” However, no data was provided to support this conclusion. The next iteration of the Beef Industry Long Range Plan (2021–2025) described traceability as “critical and urgent” to protect the health and wellbeing of US cattle (5). A goal of 75% of cattle producing states participating in a national traceability program was described in that plan. The potential utilization of improved traceability to increase beef exports and protect the domestic industry against the unmitigated spread of disease was also mentioned in the plan. The percentage of producers that adopt EID is almost certainly linked to the percentage of US beef that is traceable but the relationship between the two variables has yet to be explored. Currently there are no available quantitative estimates of the percent of electronically tagged and reported cattle needed to achieve a given level of electronic traceability.

Potential benefits from improved traceability for controlling diseases such as Foot and Mouth Disease (FMD) have been demonstrated in previous research. In an FMD outbreak model centered in the Texas high plains, Hagerman, et al. (6) found that the ability to trace 90% of movements within 2 days decreased the median number of animals that had to be slaughtered and median number of herds that were subjected to movement restrictions compared to a scenario with 30% effective tracing within 2 days. They also found that 90% effective tracing within 2 days was the lowest cost option for disease control compared to delayed or reduced efficacy tracing. In a US national FMD model with scenarios initiated in 7 different introduction sites, Smith and Sanderson found that improved tracing of approximately 70% of cattle contacts within 3 days resulted in fewer infected premises and shorter outbreak duration as compared to the baseline tracing level (7). If the tracing accuracy and speed were further increased to 99% of traces completed within 1 day, the outbreak sizes and duration could be further reduced (7). No estimate of the number of premises tagging and reporting movements to achieve those levels of traceability has been reported.

The objectives of this project were to create a simulated movement network of all cattle movements in the US, including the movements that are generally exempted from reporting, then utilize the application of electronic identification and digital movement reporting at varying probabilities to generate electronically traceable movement networks. The proportion of successfully electronically traced contacts for randomly selected cow-calf farms, feedlots, and markets in tagging and reporting scenarios was compared to the total number of contacts in the simulated full movement network.

## 2 Materials and methods

Software

All processing and analysis of simulated movement data was performed using R version 4.1.1 and R Studio 2024.09.0 (8, 9). The details of the methods described below are provided in the R code of Supplementary materials 1, 2, and 3.

### 2.1 Objective 1: network simulation

#### 2.1.1 Initialization of model

Generation of the national cattle movement network utilized the program InterSpread Plus (ISP) (Version 6.01.44) (10). ISP is a spatially explicit modeling software developed for modeling outbreaks of disease. Within a disease simulation, ISP models the risk of disease transmission via animal movements using stochastic processes to determine the source farm, the movement distance, and specific destination farm in a defined population.

The base parameter set and population file used in ISP were developed by the USDA Center for Epidemiology and Animal Health (USDA-CEAH) for the national FMD model. The geolocated population file from USDA-CEAH, which was developed from agriculture census data collected by the National Agriculture Statistics Service (NASS), includes all FMD susceptible domestic livestock species.

Movements modeled in ISP are shipments of animals from one farm to another, rather than movements of individual animals within a shipment. The rates of animal movements were defined using parameters developed from National Animal Health Monitoring System (NAHMS) studies and expert input when data was incomplete. The movements from source farms were defined by a Poisson process specific to each farm type. A list of the farm types included in the full network model is shown in Table 1. The movement distance was defined by a look-up table that included the upper bound of each movement distance interval and probability associated with that distance interval. The destination farm type was defined by a look-up table of each possible destination farm type for a given source farm and the associated probability of shipment to that destination farm type. Movements through animal markets were generated by a Poisson process at the source farm level and were defined by the final destination based on the source farm type. All movements, including those to market, were generated on a daily basis. A sample of the movement rates by farm type and region are shown in Supplementary materials 4.

**Table 1:** Farm types included in the simulated movement network and number of farms represented of each type.

Farm type	Number of farms represented in network
Small cow-calf	438,995
Large cow-calf	45,927
Small dairy heifer/calf	1,498
Medium dairy heifer/calf	1,371
Large dairy heifer/calf	474
Small dairy	29,836
Large dairy	3,451
Cattle dealer	3,031
Small feedlot	26,108
Medium feedlot	416
Large feedlot	43
Livestock market	1,098
Large stocker/backgrounder	1,870
Small stocker/backgrounder	60,688
Small processor	625
Large processor	39

In the network, farms served as nodes for the source and destination of a movement.

Over 60 movement types are defined in the USDA-CEAH ISP parameter set. The movement types relevant to this study define the direct movements of cattle from one farm to another, to markets, and to processors. The remaining movement types capture movements of other species and various aspects of indirect contact that are not relevant and were dropped in post-processing of our movement networks. ISP reports the movements in a plain-text format detailing the day of the outbreak, movement type, source farm identification, and destination farm identification. In traditional disease modeling uses, ISP only tracks and reports movements from infected farms. To coerce ISP into reporting all direct movements, all cattle farms from the national farm population file were designated as infected sites at the initiation of the model and all detection parameters deactivated. The model scenario parameters were altered to remove all other FMD control measures including movement restrictions and surveillance to generate a simulated 30-day network of all cattle movements. The completed simulated network will subsequently be referred to as the full network. See Box 1 for terminology definitions.

Box 1 **Terminology Definitions.**

Contacts: The individual farms that are identified as the result of tracing from a farm of interest.

Digital Reporting (DR): The report of a movement that is made to an accessible, official animal health database. DR used to represent the proportion of farms in the network that reported movements.

Electronic Identification (EID): The application of an electronically readable tag to a movement of animals. EID is used to represent the proportion of farms in the network that apply tags to movements.

Farm: A location where cattle are kept or congregate, may serve as a source or destination of a movement. A node in network terminology.

Farm of Interest (FOI): A cow-calf, feedlot, or market randomly selected to initiate tracing efforts.

Full Network: The network of all cattle movements generated by InterSpread Plus after post-processing.

Movements: A shipment of cattle from one farm to another. An edge in network terminology. EID/DR Scenario: The combination of and EID and DR probabilities that generated a traceable network for analysis.

Traceable Network: The resultant network after the full network was filtered to only include movements with an EID source farm and DR destination farm with the probabilities of EID and DR as defined in the EID/DR Scenario.

#### 2.1.2 Post processing of movement simulation data

##### 2.1.2.1 Market movements:

Market movements are generated on a daily basis by ISP; to represent weekly auction market sales, we aggregated 7 days of movement into the market to represent all contacts for a weekly sale. Market movements in ISP are simulated in two ways. In the first, ISP simulates markets as a “pass-through” movement where the source farm and the destination farm are specified, but not the identity of the market. Separately, ISP simulates market movements for the purposes of potential market contamination and subsequent transmission from the market. These “contamination” movements report the source farm and market identification but do not report a final farm destination.

For the purpose of tracing in our network, we required the specific origin farm, market, and destination farm to be known. For source farms making “pass-through” and “contamination” movements, we utilized the information reported for the “contamination” movements to explicitly assign a market node. If the source farm had multiple markets contacted via “contamination” movements, the closest market geographically was assigned to the “pass-through” movements. If a farm making a “pass through” movement did not complete any “contamination” movements, the market was randomly assigned within the source farm's state. Two exceptions to the same-state assignment occurred. First, farms that were in the upper peninsula of Michigan were assigned to a market in Wisconsin as opposed to simulating a shipment across Lake Michigan. Second, New Hampshire did not have any markets and Maine, Vermont, and Massachusetts had very few, so pass-through movements were assigned to a market within the combined four-state area.

##### 2.1.2.2 Model error messages: failed movements and correction:

In some cases, modeled movements “failed” in ISP and resulted in a warning message describing the attempted movement. The warning message reported the source farm identification, movement type, destination farm type, and the reason for the movement failure. Most commonly, the reason for movement failure was the inability of ISP to find the designated destination farm type within the distance interval that was selected.

The most common failed movement types were destined for a large feedlot. The ISP population only contains 43 large feedlots which are all located in the central or western states of the US. Thus, farms located outside of that area often failed to complete the generated movement because there was no large feedlot within the selected movement distance interval. The failed movements were recovered in post-processing by randomly assigning the destination to be a feedlot in the same region as the source farm, or if no such feedlot of the selected size existed in the same region (defined in supplementary material 1), then a feedlot in KS, TX, IA, NE, CO, or OK of the selected size was randomly assigned. If movements failed due to the inability to find a market, those movements were deleted from the network since no final farm destination was reported by ISP.

#### 2.1.3 Network description

The size of the full network was assessed using the total number of movements and number of farms included as nodes. The overall average degree of the network, average path length (shortest number of movements between any two farms), density, reciprocity, diameter, and transitivity were also calculated.

### 2.2 Objective 2: generation of traceable networks

#### 2.2.1 Scenario development

The baseline tagging and reporting scenario was developed using expert input from the Kansas Department of Agriculture traceability team, US CattleTrace, and other industry experts. This scenario was designed to represent the current use of EID, with most farm types being low EID adopters (1–2%) and the dairy cattle farm types having higher current utilization (95%). The probability of DR to an accessible, official animal health database was assigned to farms based on type, with most farm types low (1–5%) and dairy farm types higher (90%). Table 2 details the tagging and reporting probability used for each of the farm types in the baseline network.

**Table 2:** The tagging and reporting probability for each farm type in the baseline scenario.

Specific farm type tagging and reporting probability in baseline scenario				
Scenario name	Probability to tag (EID) outgoing movement		Probability to report (DR) incoming movement	
Baseline	S cow-calf	0.01	S cow-calf	0.01
	L cow-calf	0.02	L cow-calf	0.01
	Dairies (all)	0.95	Dairies (all)	0.9
	Stockers	0.02	Stockers	0.01
	Dealers	0.02	Dealers	0.01
	S/M feedlot	0.01	S/M feedlot	0.01
	L feedlot	0.02	L feedlot	0.05
	Market	0.1	Market	0.01
			Processor	0.02

Tagging with an EID is a characteristic applied to outgoing movements from source farms and DR is applied to incoming movements at destination farms.

The improved EID/DR scenarios were developed to assess the impact of increased adoption of EID technology and reporting by producers on traceability success. Specific combinations of increased tagging and increased reporting probabilities were developed to simulate an increased adoption of the technology at the cow-calf sector, at auction markets, or across all beef sectors. The dairy cattle EID and DR probabilities were held constant for all scenarios.

Since beef calves are born on cow-calf farms, we hypothesized that increased EID and DR probability in this farm type may impact traceability success in the feedlot and market FOIs as cattle move through the production system. Increases of EID probabilities were made for the cow-calf farms with concurrent increases in DR probabilities across all other farm types to represent how the industry as a whole might respond to the EID and DR technology being more widely adopted. Our first modeled scenario, CC 5020, increased the cow-calf EID probability to 50% and increased the DR probability to 20% for all farm types. Scenarios CC 5050 and CC 5075 maintained 50% EID usage in cow-calf farms but increased the DR in all other farm types to 50% and 75% respectively. The final cow-calf scenario, CC 7590, had 75% EID usage in cow-calf farms and 90% of all farms reporting. These scenarios are detailed below in Table 3.

**Table 3:** The tagging and reporting probability for each farm type in the cow-calf focused scenarios.

Scenario	Probability to tag (EID) outgoing movement		Probability to report (DR) incoming movement	
CC 5020	Cow-calf small and large	0.5	Dairy	Baseline
	All other farm types	Baseline	All other farm types	0.2
CC 5050	Cow-calf small and large	0.5	Dairy	Baseline
	All other farm types	Baseline	All other farm types	0.5
CC 5075	Cow-calf small and large	0.5	Dairy	Baseline
	All other farm types	Baseline	All other farm types	0.75
CC 7590	Cow-calf small and large	0.5	All farm types	0.9
	All other farm types	Baseline		

Tagging with an EID is a characteristic applied to outgoing movements from source farms and DR is applied to incoming movements at destination farms.

The change of cattle ownership at markets presents a possible site for strategic intervention in application of tags. The scenario, Market 5050, increased the EID probability of markets to 50% and increased the DR probability of all farm types to 50%. The Market 9090 scenario improved the EID and DR probability of markets to 90% and increased the DR probability to 90% for all farm types. These scenarios are detailed in Table 4.

**Table 4:** The tagging and reporting probability for each farm type in the market focused scenarios.

Scenario	Probability to tag (EID) outgoing movement		Probability to report (DR) incoming movement	
Market 5050	Markets	0.5	Dairy	Baseline
	All other farm types	Baseline	All other farm types	0.5
Market 9090	Markets	0.9	All farm types	0.9
	All other farm types	Baseline		

Tagging with an EID is a characteristic applied to outgoing movements from source farms and DR is applied to incoming movements at destination farms.

The last group of EID network scenarios were developed to assess the application of industry-wide tagging improvements. Changes in tagging and reporting were applied to all beef farm types in these scenarios. First, in the All 5075 scenario, EID probability was increased to 50% and DR to 75%. In the All 7575 scenario, EID probability increased to 75% and DR to 75%, and in the All 7590 scenario, the EID probability increased to 75% and DR to 90%. These scenarios are detailed in Table 5.

**Table 5:** The tagging and reporting probability for each farm type in the scenarios developed to reach the hypothesized 70% of movements traceable.

Scenario	Probability to tag (EID) outgoing movement		Probability to report (DR) incoming movement	
All 5075	Dairy	Baseline	Dairy	Baseline
	All other farm types	0.5	All other farm types	0.75
All 7575	Dairy	Baseline	Dairy	Baseline
	All other farm types	0.75	All other farm types	0.75
All 7590	Dairy	Baseline	All farm types	0.9
	All other farm types	0.75		

Tagging with an EID is a characteristic applied to outgoing movements from source farms and DR is applied to incoming movements at destination farms.

#### 2.2.2 Application of tagging and digital reporting

##### Step 1: assignment of tagging and reporting farms:

Sixteen cattle farm types characterize the nodes in the generated cattle network. Table 1 details the farm types and the total number of nodes in the full network. Livestock processors may only serve as a destination farm (no outgoing movements), leaving fifteen farm types to serve as sources farms for movements. EID was applied to individual source farms in a Bernoulli process *Bernoulli(p*=*probability of tagging*). Farms that were assigned to apply EID, placed tags on all cattle in outgoing movements, no partial tagging of animals within a movement was modeled. DR was applied to individual destination farms in a Bernoulli process, *Bernoulli(p*=*probability of reporting*). Farms that were assigned to DR reported all incoming tagged movements to an accessible, official animal health database.

##### Step 2: adjustment to steady-state tagging of movements:

The application of tags in step 1 does not account for tagged shipments received by non-tagging farms prior to the start of the simulated network. Shipments passing through these non-tagging farms would retain their tags as they pass through. Thus, at the steady state of the network there would be a number of tagged shipments that leave non-tagging sites. The rate of tagged, outgoing movements would be dependent on the proportion of tagged movements that had been received. For each farm that received shipments, the individual outgoing tagging probability was calculated as shown in Equation 1.


$$\begin{array}{c} outgoing\;tag\;probability=\frac{\#\;tagged\;movements\;received}{\#\;total\;movements\;received} \end{array}$$

If a farm did not receive any shipments, but did generate outgoing movements, the outgoing tagging probability for that farm was set to the average proportion of tagged movements received for that farm's type. To complete step 2, the individual movements leaving a non-tagging farm were assigned a new, “pass through” tag status in a binomial process *Bernoulli(p*=*outgoing probability)*. No changes were made to movements leaving farms that applied tags in step 1. As an example, a non-tagging farm has received and made shipments in the network. If 60% of the received shipments were tagged, then approximately 60% of the outgoing movements should be tagged. Using a binomial process *Bernoulli(p*=*0.6)*, each outgoing movement for that farm is given a tagging status in step 2. The R code for the complete tagging process for the CC 7590 scenario is detailed in supplementary materials 2.

### 2.3 Traceable network analysis

The digitally traceable networks were created by filtering the full network to only include the movements that were tagged and reported. The number of digitally traceable movements in the network represent the total number of movements that would be accessible to an animal health official for tracing, given the EID and DR probabilities of that scenario.

The traceable networks were assessed in the same metrics as the full network, described above, with the addition of the percent of movements included. In network terms, this would represent a partially observed network. The calculation for percent movements included is shown in Equation 2.


$$\begin{array}{c} Percent\;movements\;traceable\\ \;=\;\frac{\#\;movements\;in\;traceable\;network}{\#\;movements\;in\;full\;network}*100 \end{array}$$

For the remainder of this project, a movement will be defined as a shipment between two farms, and a contact as the individual farm identified in tracing efforts. As an example, two movements during the tracing period from Farm A to Farm B would only represent 1 contact for Farm B. In network terms, this would represent the unweighted presence-absence network.

#### 2.3.1 Tracing individual farms of interest (FOI)

##### 2.3.1.1 Cow-calf and feedlot FOIs:

To assess traceability, 100 randomly chosen cow-calf farms and feedlots were identified as FOIs independent of assignment to EID and DR status. The farms that were eligible for selection had at least one contact in the full network during the tracing period. The same 100 farms were used in all networks. This allowed for the estimation of total variability within a farm type to be captured within a given random generation of movements and application of EID and DR. To assess estimate variability, an additional 100 farms were selected and traced in the baseline, CC 5050 and All 7590 scenarios. Estimates after 100 and after 200 farms were compared to assess percent variation.

Since the daily nature of ISP's market movement generation does not capture the weekly intervals that most markets in the US follow, we aggregated market contacts on a 7-day basis to represent the farms that would have made contact with a market FOI during a weekly sale event. The tracing process to identify the market contacts present in the full network for cow-calf and feedlot FOIs began by identifying the movements that originated at a market and had the chosen cow-calf or feedlot FOI as the destination. If an FOI received a movement from one or more market(s), the movement file was searched, one market at a time, for the list of farms that sent a movement to the market(s) during the 7 days prior to the market's contact with the FOI. The process was completed for all markets identified for the FOI.

Direct farm-to-farm tracing was completed separately from market tracing. The tracing process for direct farm-to-farm movements began by removing all markets from the movement file. Then, the R package, EpiContactTrace, was used to trace the incoming direct farm-to-farm movements in the temporal contact chain to the FOIs (11). The function “Trace” was parameterized by the traceable network, the FOI as the starting node, model day 30 as the end day, and based on the current guidance of the USDA FMD Response Plan, the tracing period used was 28 days (12). The resultant “ingoing” direct contacts were extracted for final scenario evaluation.

The movements identified through market and direct tracing efforts were combined to determine the total incoming movements to the FOI. The combined traced movements were then converted to individual contacts by counting the unique farm identifiers present in the incoming movement record. This eliminated any duplication of movements from the same source to the FOI. This modeled process represents a starting point for disease investigation in a real-world outbreak. In the event that infected farms were identified in the tracing process, they would then become an FOI and the process would be repeated until no new infected farms were identified.

The process described above was used for tracing to the contact farms for FOIs in the full network and all derived digitally traceable networks. Figure 1 shows an example of a trace for a cow-calf or feedlot FOI. An example of this process for the cow-calf FOIs in the full network is detailed in the R code provided in supplementary material 3.

**Figure 1:**
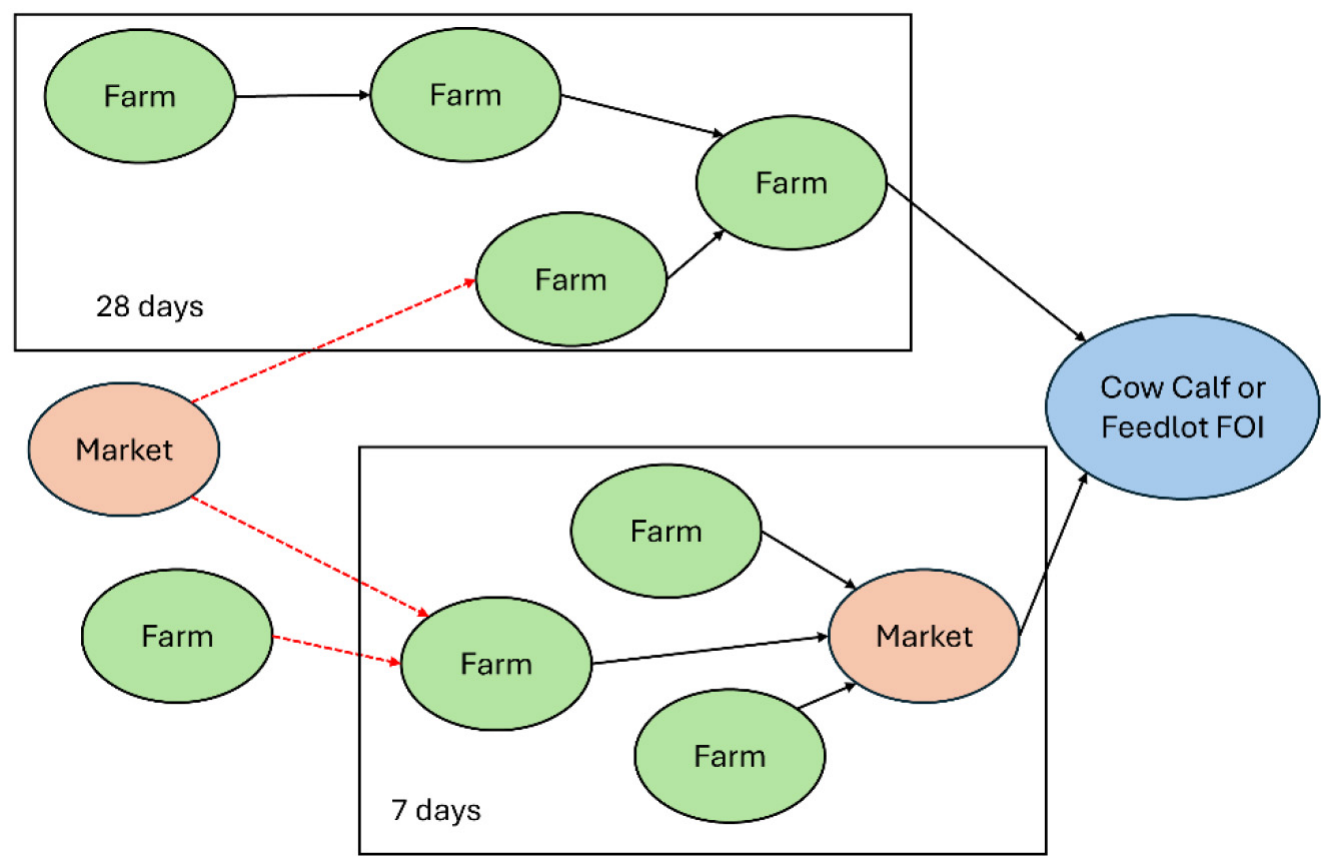
Tracing protocol for cow-calf and feedlot FOIs. If a farm or market is in a black box, it was a reported contact. Farms with red dotted arrows outside of the black box represent connections that may have been present in the model but were outside of the temporal contact scope of the tracing protocol.

##### 2.3.1.2 Market FOIs:

The selection of 100 markets as FOIs followed a similar process to the cow-calf and feedlot FOIs, but also included the selection of a random market day to begin the tracing efforts. The same 100 markets were used in all networks. Markets that received movements were filtered to only include model days 7 to 30. Then, from the list of movements filtered by day, a single random day for trace initiation was selected for each market. Finally, 100 combinations of a market and its randomly selected day were selected to be FOIs. An additional 100 markets were selected and traced in the baseline, CC 5050 and All 7590 scenarios to assess potential variability within the model. Each market FOI's direct incoming and outgoing movements were traced for the previous week as though the random day selected was the sale day that animals with clinical signs were observed at the market.

The incoming and outgoing market movements were converted to individual farm contacts. Incoming and outgoing contacts were then combined into total traceable contacts for reporting. Separate incoming and outgoing movements are included in the supplemental material 4, Figure 2 shows an example of a trace for a market FOI.

**Figure 2:**
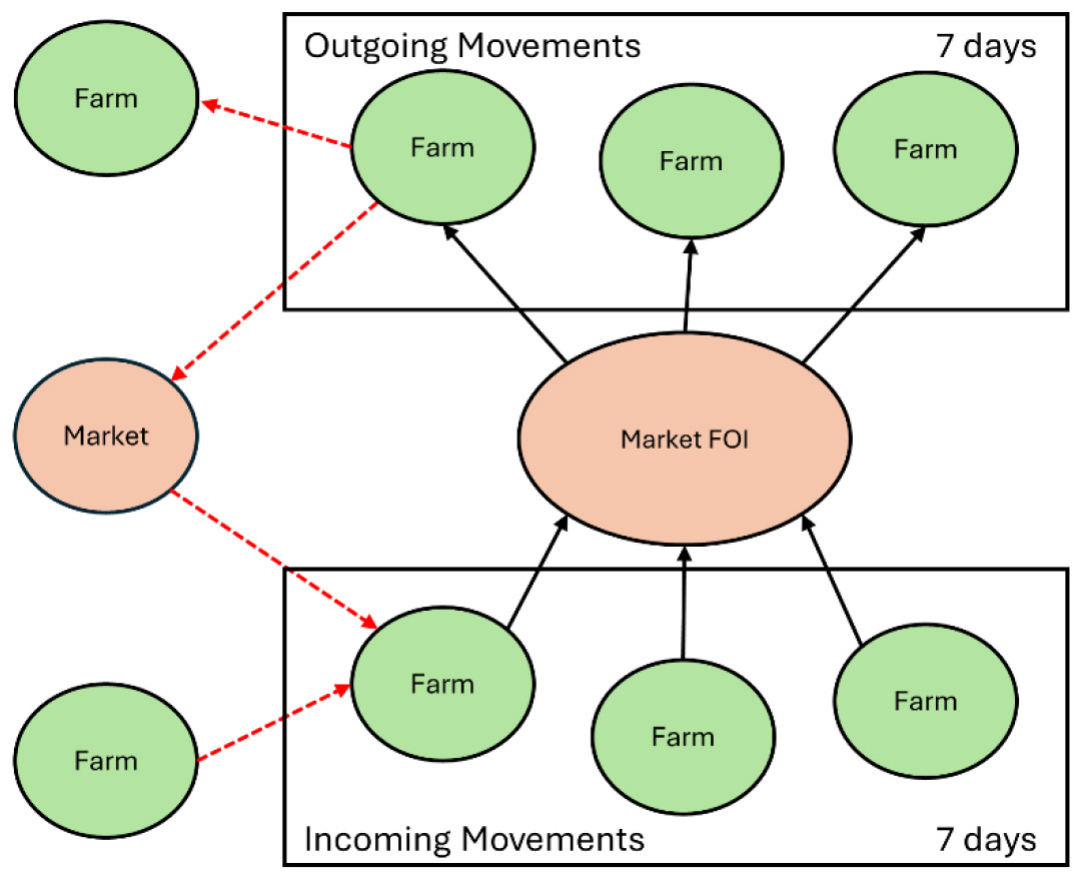
Tracing protocol for market FOIs. If a farm or market is in a black box, it was a reported contact. Farms with red dotted arrows outside of the black box represent connections that may have been present in the model but were outside of the temporal contact scope of the tracing protocol.

### 2.4 Analysis of FOI tracing outcomes

The unique farm contacts found as a result of tracing from an FOI in a traceable network was compared to the number of contacts identified for that FOI in the full network. The calculation for the percent of contacts traceable for a given FOI in a specific EID/DR scenario is shown in Equation 3.


$$\begin{array}{c} {percent\;contacts\;traceable}_{FOI,\;Scenario}\\ \;=\;\frac{\#\;contacts\;in\;traceable\;network}{\#\;contacts\;in\;full\;network}*100 \end{array}$$

## 3 Results

### 3.1 Description of the full network

The total number of movements in the full 30 day network, after all post-processing was 1,739,723. This includes 2,715 feedlot movements that were assigned a destination and 339,410 pass-through market movements that were assigned an explicit market node. A total of 392 movements were not recovered from the warning messages and were omitted representing less than 0.02% of the total movements that were included in the network. The network included 615,470 cattle farms, markets, dealers, or processors as nodes. The average path length between any two farms was 7.38 and the overall average degree (number of connections each farm has) was 5.65. The complete description of the full network metrics is shown in Table 6. Additional analysis of the full network including a matrix of movement sources and destinations is included in Supplementary material 5.

**Table 6:** Descriptive metrics for the full and traceable networks.

Scenario	Movements	% of movements traceable	Farms	Avg path length	Overall avg degree	Density	Reciprocity	Diameter	Directed transitivity
Full	1,739,723	100%	615,470	7.38	5.65	4.6 x 10^−6^	0.0108	28	0.0071
Baseline	55,180	3.17%	29,442	16.52	3.75	6.366 x 10^−5^	0.0008	51	0.0139
CC 5020	267,722	15.39%	162,796	12.03	3.29	1.010 x 10^−5^	0.0022	39	0.0033
CC 5050	578,289	33.24%	323,363	9.66	3.58	5.531 x 10^−6^	0.0041	29	0.0031
CC 5075	837,886	48.16%	426,526	8.76	3.93	4.606 x 10^−6^	0.0062	26	0.0035
CC 7590	1,234,068	70.93%	555,339	8.16	4.44	4.002 x 10^−6^	0.0069	23	0.0039
Market 5050	296,596	17.05%	90,013	9.27	6.59	3.661 x 10^−5^	0.0082	29	0.0073
Market 9090	664,580	38.20%	145,836	7.51	9.11	3.125 x 10^−5^	0.0157	22	0.0087
All 5075	1,037,027	59.61%	444,347	8.01	4.67	5.252 x 10^−6^	0.0087	24	0.006
All 7575	1,255,568	72.17%	510,253	7.81	4.92	4.822 x 10^−6^	0.0088	26	0.0061
All 7590	1,488,614	85.57%	566,505	7.56	5.26	4.638 x 10^−6^	0.0102	28	0.0065

The total number of movements (edges), farms (nodes), average path length, overall average degree, density, reciprocity, diameter, and directed transitivity for all networks.

#### 3.1.1 Number of contacts for each FOI in the full network

The range of contacts identified in the full network for cow-calf FOIs was 1 to 183 contacts with a mean of 16.26 contacts, a median of 1 contact, and 75^th^ percentile of 3.25 contacts. Of the 100 FOIs chosen, 77 had fewer than 5 contacts. Low contact numbers suggest that the FOIs had no market contacts and only limited direct farm-to-farm movements, primarily from farms that also had few incoming movements. The FOIs with higher numbers of contacts received one or more movements from market(s). The distribution of the contacts for the cow-calf FOIs is illustrated in Supplemental material 5, Figure 1.

The range of contacts for feedlot FOIs was 1 to 1,669 contacts. The mean number of contacts was 279.73, the median was 173 contacts, and 75^th^ percentile was 337.5 contacts. Out of 100 feedlot FOIs, 88 had at least one contact with a market. Many of the feedlots had multiple contacts with markets resulting in the increased number of incoming contacts compared to cow-calf FOIs. The distribution of the contacts for the feedlot FOIs is shown in Supplemental material 5, Figure 2.

The range of total contacts for market FOIs was 35 to 250 over the 7-day tracing period. The mean number of contacts was 122.26, the median was 117.5 contacts and the 75^th^ percentile was 140.25 contacts. The distribution of the total contacts for the market FOIs is shown in Supplemental material 5, Figure 3. Additional information regarding the incoming and outgoing contacts separately is also included in Supplemental material 5, Figures 4, 5.

**Figure 3:**
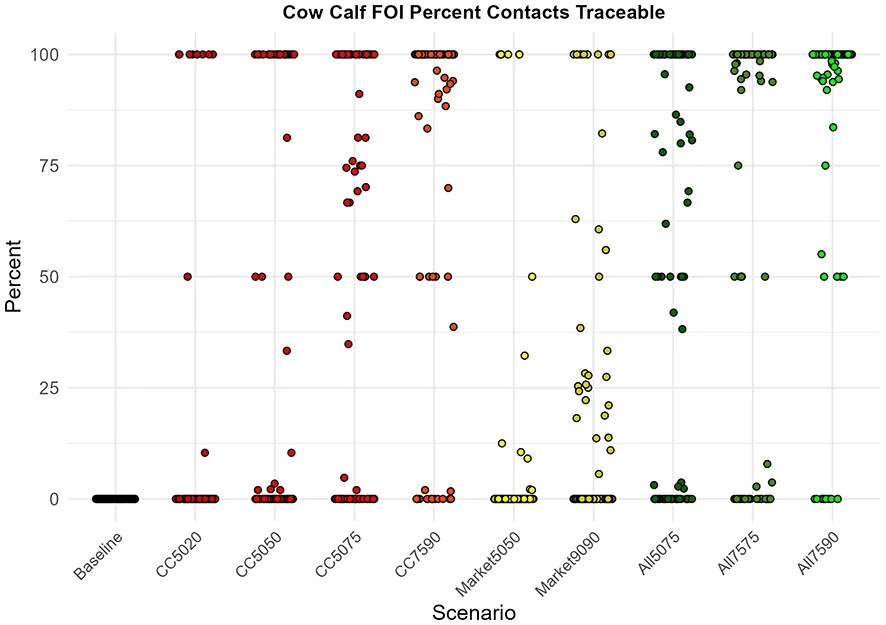
The percent of contacts from individual cow-calf FOIs that were traceable (colored circles) in each scenario.

**Figure 4:**
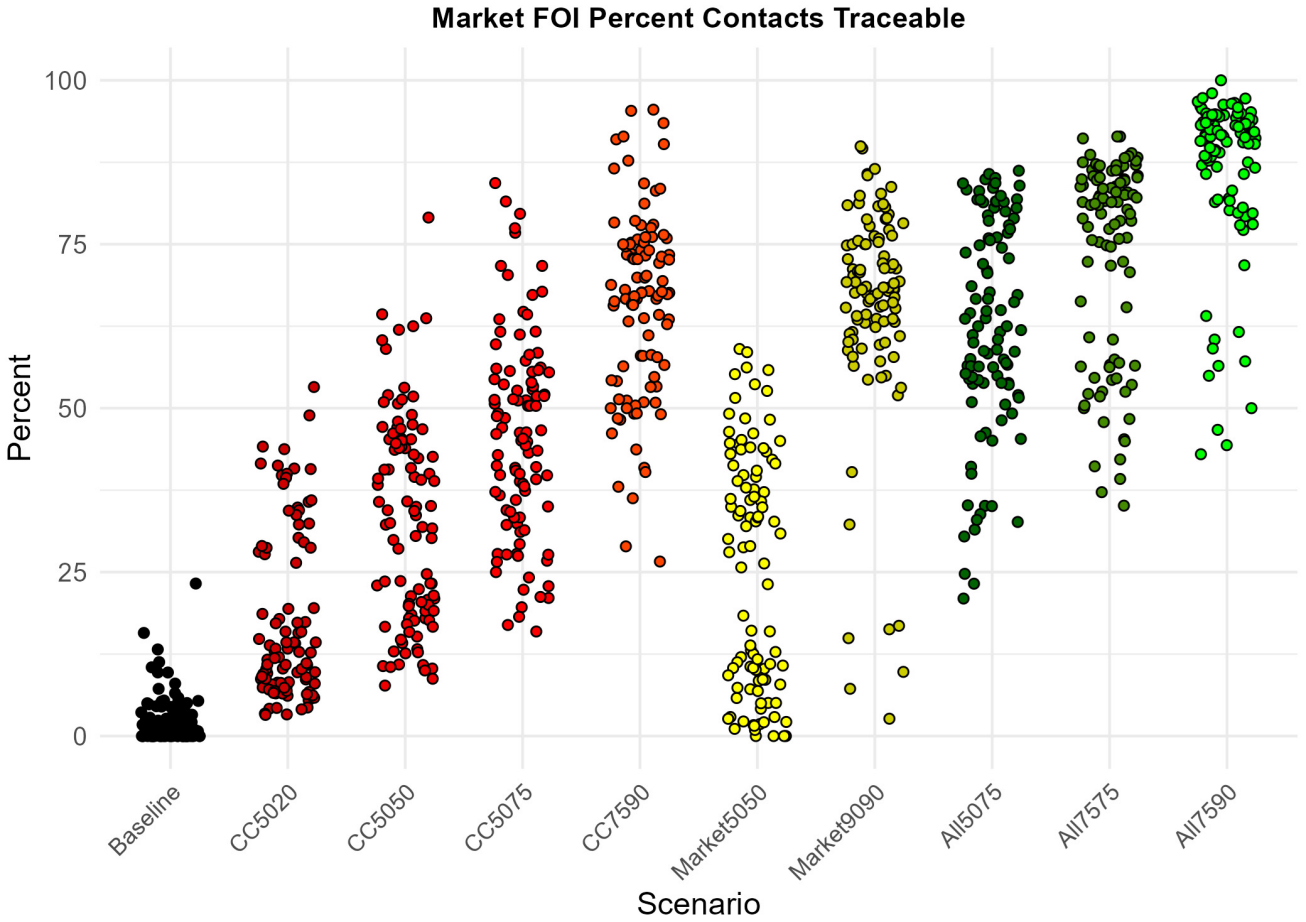
The percent of contacts from individual feedlot FOIs that were traceable (colored circles) in each scenario.

**Figure 5:**
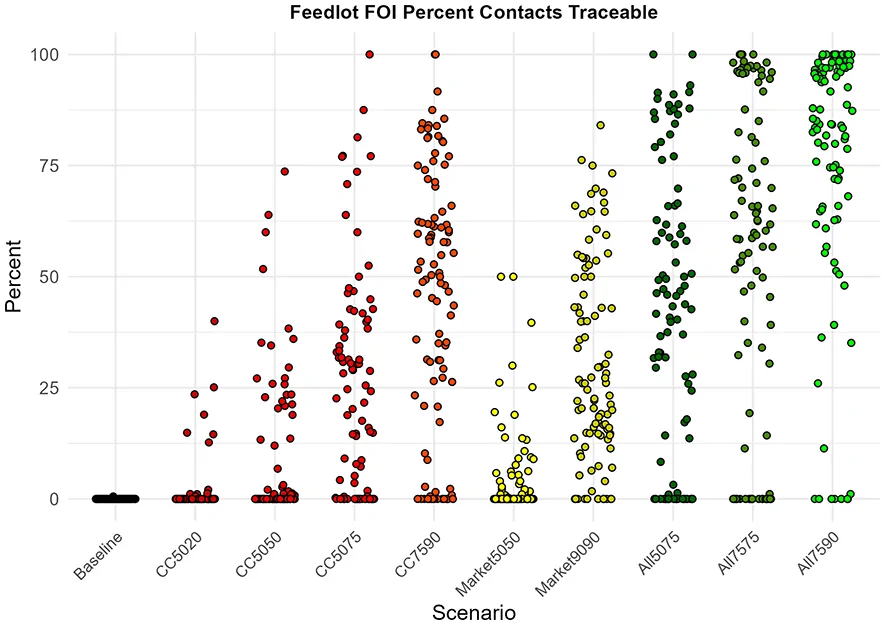
The percent of contacts from individual market FOIs that were traceable (colored circles) in each scenario.

### 3.2 Number of movements in the traceable network per scenario

The number of movements in the digitally traceable networks increased with increasing EID and DR implementation. In the baseline EID/DR scenario only 3.25% of all movements from the full network were present in the traceable network. The majority of movements were only present in traceable networks with both high probabilities of EID and DR. The most remarkable increases in number of traceable movements occurred when the EID and DR probability increases were applied to the industry as a whole. The total number of movements in each EID/DR traceable network and other network metrics are detailed in Table 6.

### 3.3 Percent of contacts traceable

The percent of contacts traceable varied widely among scenarios and FOI farm type. In the baseline scenario traceable network, a single feedlot FOI had 2 contacts traceable (of 356) while all other feedlot, cow-calf, and market FOIs had zero traceable incoming contacts. However, there were partially traceable FOIs for outgoing movements from markets at the baseline level (Supplemental material 4). The median percent of total contacts traceable for market FOIs in the baseline scenario traceable network was 2.4% with an interquartile range of 1.3% to 4.2%. The majority of the movements that were included in the baseline traceable network were dairy movements (data not shown).

The results of tracing the contact farms for FOIs in the cow-calf-focused scenario traceable networks are shown in Table 7. In the CC 5020 and CC 5050 scenario with 50% of cow-calf farms applying tags, the median percent of contacts traceable for cow-calf and feedlot FOIs did not improve from 0%. This indicates that at least 50% of the cow calf and feedlot FOIs had no traceable contacts in the CC 5020 and CC 5050 scenario networks. In the CC 5075 scenario median values of contacts traceable improved for all three FOI farm types. In the final cow-calf focused scenario, CC 7590, the cow-calf FOIs had a median of 100%, feedlot FOIs had a median of 50%, and markets FOIs had median 68% of their total contacts traceable.

**Table 7:** The mean and percentiles of percent of contacts traceable by FOI type for cow-calf-focused scenarios.

Percent contacts traceable in cow-calf-focused scenarios					
Scenario	FOI type	Mean	25th percentile	Median	75th percentile
CC5020	Cow-calf	8.60%	0.00%	0.00%	0.00%
	Feedlot	1.56%	0.00%	0.00%	0.00%
	Market	17.39%	7.75%	11.96%	28.25%
CC5050	Cow-calf	31.85%	0.00%	0.00%	100.00%
	Feedlot	7.65%	0.00%	0.00%	4.05%
	Market	32.70%	18.92%	33.07%	44.75%
CC5075	Cow-calf	49.33%	0.00%	50.00%	100.00%
	Feedlot	22.36%	0.00%	15.57%	36.68%
	Market	45.76%	34.39%	46.16%	55.49%
CC7590	Cow-calf	75.16%	50.00%	100.00%	100.00%
	Feedlot	45.16%	22.71%	50.18%	64.96%
	Market	66.07%	55.98%	67.59%	75.00%

In the market-focused scenarios, the cow-calf FOIs had a median of 0% traceable contacts in both scenarios and only 24% of contacts were traceable at the 75^th^ percentile in the Market 9090 scenario. Feedlot FOIs had a median 0% traceable contacts in the Market 5050 scenario, but in the Market 9090 scenario achieved a median of 25.29% of contacts traceable. The market median total contacts traceable was 28.41% in the Market 5050 scenario and 68.2% the Market 9090 scenario. The results of the market-focused scenarios are detailed in Table 8. The market FOIs' increased success was especially notable in the outgoing movement tracing in the market-focused scenarios (Supplementary material 4).

**Table 8:** The mean and percentiles of percent of contacts traceable by FOI type for market-focused scenarios.

Percent contacts traceable in market-focused scenarios					
Scenario	FOI type	Mean	25th percentile	Median	75th percentile
Market 5050	Cow-calf	7.19%	0.00%	0.00%	0.00%
	Feedlot	4.19%	0.00%	0.00%	2.87%
	Market	25.08%	8.54%	28.41%	40.67%
Market 9090	Cow-calf	18.92%	0.00%	0.00%	24.43%
	Feedlot	30.35%	14.58%	25.29%	49.79%
	Market	65.5%	61.5%	68.2%	75.1%

When the EID and DR probabilities were improved for all beef-cattle farms, the success of tracing contacts for all FOI types increased notably. In the All 5075 scenario, the feedlot and market FOIs had median values of 41.24% and 62.2% of contacts traceable then increased to 59.01% and 81.09% in the All 7575 scenario. The cow-calf FOIs had median values that began at 73.62% in the All 5075 scenario and 100% in the All 7575 and All 7590 scenarios. Despite the high median values, the mean value for cow-calf FOIs started at 55.02% of contacts traceable in the All 5075 scenario and increased to 83.56% in the All 7590 scenario. These results are detailed in Table 9.

**Table 9:** The mean and percentiles of percent of contacts traceable by FOI type for industry-wide scenarios.

Percent contacts traceable in all beef-focused scenarios					
Scenario	FOI type	Mean	25th percentile	Median	75th percentile
All 5075	Cow-calf	55.02%	0.00%	73.62%	100.00%
	Feedlot	40.01%	0.00%	41.24%	63.52%
	Market	62.3%	53.8%	62.2%	77.5%
All 7575	Cow-calf	65.95%	0.00%	100.00%	100.00%
	Feedlot	52.06%	0.83%	59.01%	85.66%
	Market	73.67%	59.71%	81.09%	84.91%
All 7590	Cow-calf	83.56%	94.66%	100.00%	100.00%
	Feedlot	75.63%	65.61%	84.30%	96.93%
	Market	86.5%	85.1%	91.4%	93.9%

The individual FOI percent of contacts traceable per scenario are plotted in Figures 3–5 for each FOI farm type. The distribution of number of contacts in the full network by cow-calf FOIs was influential in the distribution of possible contacts traceable. Specifically, for 59 of the cow-calf FOIs there was only 1 contact in the full network, which results in a binary outcome of 0% or 100% traceability in the traceable networks. This relationship is illustrated in Figure 3 where a large portion of the data points are at the extremes of the graph. The feedlot FOIs were more widely variable in their possible results and showed a clear increase in success of tracing with improved EID and DR probabilities. However, across all improved EID/DR scenarios, there are individual cow-calf and feedlot FOIs that were completely untraceable and remained at 0% of contacts found. The individual feedlot FOIs are illustrated in Figure 4. The market FOIs were the only FOI type to achieve at least partial traceability for all FOIs in all scenarios excluding Baseline and Market 5050. This is illustrated in Figure 5 where there are no data points at 0%. Across the scenarios, the increased tracing success with increased EID and DR implementation is visible.

### 3.4 Assessment of variability

The change in estimates from tracing 100 farms and 200 farms for each farm type that were traced in the baseline, CC 5050 and All 7590 had less than 5% change across all three traced in percent contacts found from the reported 100 farm types. Median difference from 100 to 200 farm traces ranged from 0% in scenarios with 0% traceability, to 3.2% for Markets in the CC 5050 scenario. Based on this evidence of estimate convergence we report results for the 100 farm estimates.

## 4 Discussion

In this study we created a simulated movement network for all cattle movements in the US, and generated traceable networks using EID tagging and digital reporting of movements. The traceability of contacts of randomly selected farms was compared between the traceable networks. The network-level results of this study saw an improvement from 3.17% of total movements being present in the traceable network under the current estimated use of EID and DR to 85.57% of movements present in the traceable network under the highest EID and DR probabilities modeled. The baseline scenario serves as a possible estimate of the current digital traceability of cattle in the US. The actual value may be different but likely remains much lower than the EID and DR scenarios that were modeled in this study. Our analysis is focused on possible achievable levels of traceability rather than improvement from baseline. The scenarios that focused on improving EID implementation at cow-calf or market origins made small to modest improvements in the traceability of contacts for all FOI types. The most remarkable improvements were seen in the scenarios All 7575 and All 7590 where all farm types had increased EID and DR probabilities.

The reported range of percent contacts traceable represents the possible success for tracing the contacts of a given farm type. This value is the estimated traceability success that an animal health official or regulator can expect with the improved implementation of EID and DR in each scenario. We used current estimates of EID and DR use probabilities in the cattle industry obtained from animal health regulatory officials and other industry experts to parameterize the baseline traceable network. The experts consulted by the authors expressed that currently, digital tracing often has a low probability for success, which was consistent with the poor performance observed for our baseline scenario.

The necessary speed for tracing depends on the disease being traced. For example, bovine tuberculosis is slowly transmitted throughout a population so rapid tracing is less critical; whereas, FMD is rapidly transmitted between animals and herds and rapid tracing of contacts to implement surveillance and control is needed. The higher levels of EID and DR use modeled in our scenarios are likely necessary to achieve timely digital tracing success during an FMD outbreak. Furthermore, EID and DR use needs to increase simultaneously to result in effective digital tracing.

In an actual FMD outbreak, the 28 days of trace-back efforts would be initiated at the time clinical signs are detected within a herd (12). To reflect this practice, we modeled trace back efforts for the 28 days prior to day 30 of the simulated network for each cow-calf and feedlot FOI. Additionally, our methods reflect tracing the contacts that may have occurred as the direct result of a weekly livestock auction. This practice of tracing 28 days of direct farm contacts and 1 week of auction market contacts reflects a resource-conscious method of tracing that would likely be implemented in a disease outbreak. In the event that a positive farm is identified during the trace, that farm would become a new FOI with 28 days of trace-back efforts being repeated for the new FOI. Additionally, in an outbreak, forward tracing would also be done to identify farms where the FOI may have sent potentially infected cattle. While we did not model forward tracing from cow-calf and feedlot FOIs, the tracing process and tagging probabilities within the traceable network would remain the same and would result in similar traceability results to those reported here.

Trace-back efforts from cow-calf and feedlot FOIs identified the traceability of contact farms that were directed into that farm type. The cow-calf FOIs tended to have the lowest success in identifying contacts in market-focused scenarios. This is likely due to a majority of tags being applied after cattle had left the cow-calf site and made little difference on incoming contact traceability. Our process assumes tagging markets apply tags to all cattle in outgoing shipments and reporting markets report incoming tagged shipments. If cattle in an incoming shipment were not tagged, it was not digitally traceable (not present) in our traceable networks. Our results suggest that tagging implementation in only markets may not result in high digital traceability to cow-calf farms unless the market bears the burden to completely report the source of cattle regardless of previous tagging status. In comparison, the traceability of feedlot FOIs improved more than the cow-calf FOIs when the market EID and DR probabilities were improved.

Access to movement data by regulatory personnel is critical for rapid tracing, so tags must be applied to cattle and movements must be reported to an accessible, official database. For this project we assumed that all movements were reported to a database that was completely accessible to regulatory officials conducting the traces. Currently, there is not a comprehensive, centrally accessible database for livestock movement in the US. Instead, the US has multiple databases that may contain pieces of the information needed to conduct a trace. The Animal Identification Management System (AIMS) contains the animal identification numbers allocated to manufacturers and to whom the tags were distributed (13). This database may contain contact and premises information for the individual producer or the last distributor of the tag, such as the accredited veterinarian. It is the responsibility of the accredited veterinarian to maintain the final record of tag application in an accessible system (13). Each state can utilize a database of their choosing to manage various animal health programs, traceability, and other data. There is some overlapping information in the databases that states may choose, but many states do not use the same database(s) for all information. In some states, the electronic certification of veterinary inspection (CVI) programs can retrieve the owner and animal identification data directly from the accredited veterinarian's submission form, if a CVI was submitted for the movement. To improve inter-state data availability, the Federal Animal Health Events Repository (AHER) maintains electronic records of animal health events including animal disease traceability data (14). States may choose to submit records of location and date of observation of animals with official identification to the AHER database. A reported observation of an animal in the AHER database can allow an animal to be traced to its last known state. To improve the efficiency of digital tracing, states would need to agree on what system will be used and how data will be input and applied, while maintaining data security and privacy of animal producers. A possible solution would be a central database that houses the location (state or private database) of specific movement records.

The results reported here reflect the percent of contacts that could be identified within 1 day using electronic methods, and do not represent any additional methods of tracing or a timeline for successful completion of non-electronic traces. Achieving high traceability of cattle during non-disease outbreak periods is necessary to high cattle traceability during an outbreak. Previously Smith and Sanderson found that in addition to stamping out and movement controls, tracing and identifying approximately 23% of movements within 1 day, 52% within 2 days, and 76% within 3 days could be impactful in controlling an FMD outbreak (7). Based on those study results, the nominal changes in electronic tracing success made in our scenarios with 50% of cow-calf farms tagging may be insufficient to effectively control an FMD outbreak. While manual tracing efforts are effective, they may not be rapid enough to impact the progression of an FMD disease outbreak. However, in several scenarios, the median and mean percent of contacts traceable for all farm types approached or exceeded 50% and may reflect a level of tagging and reporting that could improve FMD disease control. As a whole, the goals for EID and DR adoption in the US may need to be higher than previously estimated in the Beef Long Range plan (5) and World Perspectives report (4) to be effective for aiding disease control on all farms in an outbreak scenario in the US.

Generally, the US beef cattle sector has been reluctant to adopt official EID and DR technology. Cost of tags and the concern for data privacy and security is likely influencing some of the reluctance. In response, the USDA has programs for producers to receive tags at no-cost through their state veterinarian office and has shared documents for producers stating that the confidentiality of the identifying information in the movement data is protected by law and such information is only accessible to officials that have security permissions and are conducting a disease trace (15). Additionally, the majority of beef cattle are exempt from current legal requirement to have an official form of identification that is both visually and electronically readable (16, 17). The current ADT rule does not include non-dairy cattle under 18 months of age or those that have been sexually altered (spayed heifer/steer) (3). Under the current system, a beef animal may have unofficial identification and privately held records of ownership and movement which would not be accessible to animal health officials in the event of an outbreak.

Our study results demonstrate the performance of electronic tracing systems alone, without the implementation of other tracing methods. Ultimately, success of a digital trace is dependent on the continued presence of digital connections from farm to farm in the incoming movement chain; if a digital connection is missing, there is no ability to identify the prior incoming contact(s). The failure of the first movement to be traceable was seen across all scenarios, including those with industry-wide, high levels of EID and DR. In an outbreak, a producer is likely to be able to manually report their direct contacts to a surveillance team and ensure the connection to the immediately previous source farm or market can be identified, which would allow the initiation of an electronic trace from the identified premises. Our discussion with State Animal Health Officials suggested that making phone calls to producers was commonplace in the current tracing efforts and would easily be implemented in an outbreak scenario. Additionally, in many states, markets are required to keep records of incoming animals and routine reviews of those records are conducted for compliance. Using all available data sources, both digital and analog, would result in higher tracing success than relying on digital data sources alone, though tracing would not be completed as rapidly. Further, while rapid tracing is part of an effective outbreak response it would not replace other measures to bring the outbreak under control.

Our simulated network generated movements utilizing random processes that may not capture the explicit trade networks that exist within the US cattle industry. We believe our simulated network captures reasonable estimates of shipment rates between production types and is fit for the purpose of assessing tracing of the contacts of a selected FOI. The results of the study are not dependent on a specific network but rather on representation of the network connections between beef production types. This simulated network is different from other studies which have utilized the collection of real movement data but are often limited to movements on a state, county, or grid scale and do not include within state movements or movements directly to slaughter (18, 19). Both Cabezas, et al. (18), and Buhnerkempe, et al. (19), utilized Interstate Certificates of Veterinary Inspection (ICVI) to estimate the number of movements that occurred with some inference from the ICVI data to estimate data gaps. Our full network includes within-state movements that do not require an ICVI and, therefore, would not have been included in previous research efforts. Additionally, the network in this study included movements to and from livestock markets.

The simulated network reported here does not model the movement of individual animals, only the connection from source to destination. Because of this, one of our model assumptions was that tags were applied to entire movement groups. Additionally, EID and DR status of farms were fixed attributes, so farms did not change their tagging status during the modeled 30-day period. In the 30-day sample of movements that were used, this assumption is reasonable. Realistically, a cow-calf producer may ship their calf crop in one large sale lot to a market once per year. Then they might also take multiple small shipments of cows or bulls to be culled to market. Currently, for interstate movements, the calves would be exempt from individual identification but the cows and bulls would be required to have an individual, official EID tag. Our current model does not capture those unique EID requirement variations in shipments from one farm, but does capture the effects of overall increased tagging and reporting across shipment types regardless of intrastate or interstate movement. Further, in actuality, some animals in a shipment may not have ever been tagged or may have lost their tag. If these cattle are then sorted into different shipments at a market, separating them from their original group, without individual identification, these cattle become untraceable. Our method does not capture this potential.

The applications of improving traceability are not limited to animal disease traceability and outbreak control. Producers could leverage the verifiable history of their animals to achieve added value at market through product labels that reflect location of origin, production practices, or other characteristics desirable to consumers. The increase in consumer demand for knowledge about the products they are consuming can be readily captured by improving information sharing through traceability systems. Those benefits can be realized by the individual producer that applies the tags and tracks the data, however industry-wide benefits of rapid disease tracing in the case of an FMD outbreak will require implementation by a high percentage of farms. Other industry benefits could be improved value in export markets to countries that recognize traceability as an asset.

## 5 Conclusion

With this study, we showed that the current estimated usage of EID and DR in the US beef cattle industry resulted in digital tracing failure for all three FOI types, supporting the conclusion that the current level of implementation for these technologies is not effective for completing electronic-only animal disease traces during an outbreak of a rapidly transmitted disease such as FMD. In our 30-day network, increasing cow-calf EID tagging to 75% and digital reporting to 90% (CC 7590) resulted in median trace success greater than 50% for all three FOI types. Median contact tracing success in scenarios where EID use in all cattle farm types was increased to 50% with 75% reporting (All 5075) approached or was greater than 50% for all FOI types. When EID was implemented for 75% of all cattle farm types and DR was increased to 90% (All 7590), the median contact tracing success was greater than 84% for all FOI types, which could be highly impactful in outbreak control. These results do not reflect individual animal traceability but do support the need for not only improving EID use but also a need for high DR into accessible databases. The value of EID use for producers can extend beyond disease control and be applied to potential value-added marketing of their products and on-farm data management. Future work could incorporate individual animal EID and DR within a given farm to assess the impact of partially tagged movements on traceability success. Success of an electronic tagging program in cattle will be dependent on more than its utility in disease tracing. Long term success must return value for producers. As such more research is also needed to explore the economic trade-offs, costs, and market benefits to support the adoption of this technology at the national scale.

## Data Availability

The raw data supporting the conclusions of this article will be made available by the authors, without undue reservation.
