# Supplementary material for: Assessment of cattle traceability using electronic identification and digital movement reporting in simulated United States cattle movement networks
Source: Front Vet Sci. 2026 Oct 27;13:1972037. doi: 10.3389/fvets.2026.1972037 (PMC13747793; doi:10.3389/fvets.2026.1972037)
Supplement: Supplementary file 1 [file Data_Sheet_1.PDF]

Post Processing of Movement Network

Definitions:
Movement File: The output from ISP
Irrelevant species: non-cattle source or destination farms
Easting/Northing: The X and Y coordinate system used by ISP for farm geolocation

The purpose of this code is to generate the movement networks that can be assessed for traceability.
Step 1: Remove all non-cattle and indirect movements from the ISP output.
Step 2: Assign the movements that passed through a market a specific market location
Step 3: Recover the movements that failed from the warning file and assign them a destination.

The original simulation was for 60 days of movements throughout this code, the movements will be filtered to 30 days.

```
library(dplyr)

library(tidyverse)

FarmFile<-read_csv("C:/Users/marykasmith/Desktop/New FF and Markets/FarmFileStates2024
.csv")

FarmFile<-FarmFile%>%select(-...1)

Movements<-read.table("C:/Users/marykasmith/Desktop/60d Run 2/Movements60d.2.txt")
```

Begin by adding the farm types back into the source and destination farms

```
colnames(Movements)<-c("Iteration","DayMov","Source","EastingS","NorthingS", "Destinat
ion","EastingD","NorthingD","Type")

Movements<-Movements%>%filter(DayMov<31)

FarmFile_Source<-FarmFile%>%select(FarmID, Class,State)

FarmFile_Source<-FarmFile_Source%>%rename(Source=FarmID, S.FarmType=Class, S.State=Sta
te)


MovementSources<-left_join(Movements,FarmFile_Source,by="Source")


FarmFile_Dest<-FarmFile%>%rename(Destination=FarmID, D.FarmType=Class, D.State=State)

MovementsComplete<-left_join(MovementSources,FarmFile_Dest,by="Destination")


MovementTable<-table(MovementsComplete$Type) #Optional to see movement Types
```

```
print(MovementTable)

table(MovementsComplete$DayMov) #optional to confirm all days present
```

Remove movement types that are indirect movements that are in the model parameters for capturing the contamination of markets or other locations

```
ReMoveIndirect<-MovementsComplete%>%filter(Type!="MovementType60"&
                                Type!="MovementType61"&
                                Type!="MovementType62"&
                                Type!="MovementType63"&
                                Type!="MovementType64"&
                                Type!="MovementType54"&
                                Type!="MovementType55"&
                                Type!="MovementType56"&
                                Type!="MovementType57"&
                                Type!="MovementType58"&
                                Type!="MovementType59")
#table(ReMoveIndirect$S.FarmType) #optional to see farm types represented
```

Remove all sources that are not cattle and the odd cattle farm that ships to bison processors

```
OnlyCattle<-ReMoveIndirect%>%filter(S.FarmType!="bison"&
                                S.FarmType!="dealer_bison"&
                                S.FarmType!="transitional_swine"&
                                S.FarmType!="sheep_s"&
                                S.FarmType!="sheep_l"&
                                S.FarmType!="other_swine_s"&
                                S.FarmType!="other_swine_l"&
                                S.FarmType!="nursery_s"&
                                S.FarmType!="nursery_l"&
                                S.FarmType!="grower_finisher_l"&
                                S.FarmType!="grower_finisher_s"&
                                S.FarmType!="livestock_market_goat_sheep"&
                                S.FarmType!="livestock_market_swine"&
                                S.FarmType!="dealer_goat"&
                                S.FarmType!="dealer_sheep"&
                                S.FarmType!="dealer_swine"&
                                S.FarmType!="farrow_to_feeder_l"&
                                S.FarmType!="farrow_to_feeder_s"&
```

```
                                    S.FarmType!="farrow_to_finish_l"&
                                    S.FarmType!="farrow_to_finish_s"&
                                    S.FarmType!="farrow_to_wean_l"&
                                    S.FarmType!="farrow_to_wean_s"&
                                    S.FarmType!="goat_dairy"&
                                    S.FarmType!="feedlot_sheep"&
                                    S.FarmType!="goat_other"&
                                    D.FarmType!="processor_bison")

head(OnlyCattle)#optional to confirm only cattle are present

CattleSource<-as.data.frame(table(OnlyCattle$S.FarmType)) #optional to see all sources of cattle

CattleDestinations<-as.data.frame(table(OnlyCattle$D.FarmType)) #optional to see all destinations cattle shipped to
```

This completes the removal of indirect movements and irrelevant species.

Currently, MovementType42 does not have a specific market node that they are passing through. This will be remedied at the end of the clean up after the movements that were in the warning file as “can not find destination farm” are re-incorporated into the movement file. These failed movements are typically the result of the inability to find a farm of the desired type in the distance band that ISP selected. In reality, a producer would just ship farther.

At this time, there are several dataframes created that are not needed anymore and can be removed for space

```
rm(FarmFile_Dest, FarmFile_Source, ReMoveIndirect, Movements, MovementsComplete, MovementSources, MovementTable); gc()
```

Bringing in the movements from the warning file - The warning file has been pre-edited in Notepad++ to “mark” all of the lines that had “can not find destination farm” and those were copied to a new text file. Then all lines that included “indirect” were marked and removed.

The following code could likely be simplified by more pre-editing of columns in the plain-text files

```
DestinationWarning<-read.table("C:/Users/marykasmith/Desktop/60d Run 2/Cannot find destination farm 60d2.txt", sep="\t")

head(DestinationWarning)

DestinationWarning<-DestinationWarning%>%select(V5,V9,V11) #Day, MovementType, column with destination information

DestinationWarning<-DestinationWarning%>%separate(V11, into = c("A", "B","C","D"), sep = "=") #Placeholders to separate out the line lines of text

DestinationWarning<-DestinationWarning%>%select(-A, -D)

DestinationWarning<-DestinationWarning%>%separate(B, into = c("Nada","FarmID", "5","6","7","8","9"), sep = " ")

DestinationWarning<-DestinationWarning%>%separate(C, into = c("DestClass", "Other"), sep = ",")
```

```
DestinationWarning<-DestinationWarning%>%select(V5, FarmID, DestClass,V9)

DestinationWarning<-DestinationWarning%>%rename(DayMov=V5)

DestinationWarning <- transform(DestinationWarning,
                             FarmID = as.numeric(FarmID))
```

Assess what movement types are present and change them to the same system as the original output file

```
DestWarnSource<-left_join(DestinationWarning, FarmFile, by="FarmID")

DestWarnSource<-DestWarnSource%>%rename(S.FarmType=Class, EastingS=Easting, NorthingS=
Northing)

DestWarningOnlyCattle<-DestWarnSource%>%filter(S.FarmType!="bison"&
                                S.FarmType!="dealer_bison"&
                                S.FarmType!="transitional_swine"&
                                S.FarmType!="sheep_s"&
                                S.FarmType!="sheep_l"&
                                S.FarmType!="other_swine_s"&
                                S.FarmType!="other_swine_l"&
                                S.FarmType!="nursery_s"&
                                S.FarmType!="nursery_l"&
                                S.FarmType!="grower_finisher_l"&
                                S.FarmType!="grower_finisher_s"&
                                S.FarmType!="livestock_market_goat_sheep"&
                                S.FarmType!="livestock_market_swine"&
                                S.FarmType!="dealer_goat"&
                                S.FarmType!="dealer_sheep"&
                                S.FarmType!="dealer_swine"&
                                S.FarmType!="farrow_to_feeder_l"&
                                S.FarmType!="farrow_to_feeder_s"&
                                S.FarmType!="farrow_to_finish_l"&
                                S.FarmType!="farrow_to_finish_s"&
                                S.FarmType!="farrow_to_wean_l"&
                                S.FarmType!="farrow_to_wean_s"&
                                S.FarmType!="goat_dairy"&
                                S.FarmType!="feedlot_sheep"&
                                S.FarmType!="goat_other"&
```

```
                                  S.FarmType!="processor_swine")
DestWarningOnlyCattle<-DestWarningOnlyCattle%>%mutate(Type = case_when(
    V9 == "cattle_dealer"  ~ "MovementType19",
    V9 == "cattle_market" ~ "MovementType42",
    V9 == "small_cattle_feedlot" ~ "MovementType13",
    V9 == "large_medium_cattle_feedlot" ~ "MovementType12",
    V9 == "large_cow_calf_MW_PC" ~ "MovementType3",
    V9 == "large_cow_calf_GL_NE_SE" ~ "MovementType4",
    V9 == "small_cow_calf_MW_PC" ~ "MovementType5",
    V9 == "small_cow_calf_GL_NE_SE" ~ "MovementType6",
    V9 == "large_dairy" ~ "MovementType7",
    V9 == "small_dairy" ~ "MovementType8",
    V9 == "large_dairy_heifer_calf" ~ "MovementType9",
    V9 == "medium_dairy_heifer_calf" ~ "MovementType10",
    V9 == "small_dairy_heifer_calf" ~ "MovementType11",
    V9 == "large_stocker_GL_MW_NE_PC" ~ "MovementType14",
    V9 == "large_stocker_SE" ~ "MovementType15",
    V9 == "small_stocker_GL_MW_NE_PC" ~ "MovementType16",
    V9 == "small_stocker_SE" ~ "MovementType17",
    TRUE                           ~ NA_character_
  ))
table(DestWarningOnlyCattle$Type)


table(DestWarningOnlyCattle$S.FarmType)
```

There are many NA types in the dataframe at this point. They are all indirect or other movement types we don't care about. They'll be removed now.

```
DestWarningOnlyCattle <- DestWarningOnlyCattle %>%
  filter(if_all(everything(), ~ !str_detect(., regex("farrow", ignore_case = TRUE))))
DestWarningOnlyCattle <- DestWarningOnlyCattle %>%
  filter(if_all(everything(), ~ !str_detect(., regex("sheep", ignore_case = TRUE))))
DestWarningOnlyCattle <- DestWarningOnlyCattle %>%
  filter(if_all(everything(), ~ !str_detect(., regex("swine", ignore_case = TRUE))))
DestWarningOnlyCattle <- DestWarningOnlyCattle %>%
  filter(if_all(everything(), ~ !str_detect(., regex("goat", ignore_case = TRUE))))
DestWarningOnlyCattle <- DestWarningOnlyCattle %>%
  filter(if_all(everything(), ~ !str_detect(., regex("finish", ignore_case = TRUE))))
```

```
DestWarningOnlyCattle <- DestWarningOnlyCattle %>%
  filter(if_all(everything(), ~ !str_detect(., regex("bison", ignore_case = TRUE))))
DestWarningOnlyCattle <- DestWarningOnlyCattle %>%
  filter(if_all(everything(), ~ !str_detect(., regex("nursery", ignore_case = TRUE))))
DestWarningOnlyCattle <- DestWarningOnlyCattle %>%
  filter(if_all(everything(), ~ !str_detect(., regex("indirect", ignore_case = TRUE)))
)
DestWarningOnlyCattle <- DestWarningOnlyCattle %>%
  filter(if_all(everything(), ~ !str_detect(., regex("infect", ignore_case = TRUE))))

table(DestWarningOnlyCattle$S.FarmType) #optional to confirm only cattle
table(DestWarningOnlyCattle$DestClass) #to see where they are trying to go
```

All the farms now need to be a matched to a destination farm. The feedlots will be in KS, TX, or CO. All others will just be randomly assigned to a destination in their region.

```
DestWarningOnlyCattle<-DestWarningOnlyCattle%>%rename(S.State=State)
DestWarningOnlyCattle<-DestWarningOnlyCattle%>%filter(DayMov<31)
FarmFileDest<-FarmFile%>%rename(
  Destination=FarmID,
  D.FarmType=Class,
  EastingD=Easting,
  NorthingD=Northing,
  D.State=State
)
```

Create the regions

```
GreatLakes<-c("Minnesota","Iowa","Missouri","Indiana","Ohio","Wisconsin","Michigan","Kentucky","Illinois")

MountainWest<-c("Montana","North Dakota","South Dakota","Idaho","Utah","Nevada","Colorado","Arizona","New Mexico","Texas","Nebraska","Oklahoma","Kansas","Wyoming")

Northeast<-c("Maine","New Hampshire","Vermont","Massachusetts","Rhode Island","Connecticut","New Jersey","Delaware","Maryland", "West Virginia","Virginia","New York","Pennsylvania")

Pacific<-c("California","Oregon","Washington")
```

```
Southeast<-c("Arkansas","Louisiana","Mississippi","Tennessee","Alabama","Georgia","Florida","North Carolina","South Carolina")


Feedlot<-c("Kansas","Texas","Colorado","Iowa","Nebraska","Oklahoma")
```

Assign the movements to a region based on the source state and the farm file

```
DestWarningOnlyCattle<-DestWarningOnlyCattle%>%mutate(Region = case_when(
  S.State %in% GreatLakes ~ "GreatLakes",
  S.State %in% MountainWest ~ "MountainWest",
  S.State %in% Northeast ~ "Northeast",
  S.State %in% Pacific ~ "Pacific",
  S.State %in% Southeast ~ "Southeast"))

FarmFileDest<-FarmFileDest%>%mutate(Region = case_when(
  D.State %in% GreatLakes ~ "GreatLakes",
  D.State %in% MountainWest ~ "MountainWest",
  D.State %in% Northeast ~ "Northeast",
  D.State %in% Pacific ~ "Pacific",
  D.State %in% Southeast ~ "Southeast"))

FarmFileDest<-FarmFileDest%>%mutate(RegionB = case_when(
  D.State %in% Feedlot ~ "Feedlot"))
Feedlot_s<-FarmFileDest%>%filter(D.FarmType=="feedlot_cattle_s")
Feedlot_m<-FarmFileDest%>%filter(D.FarmType=="feedlot_cattle_m")
Feedlot_l<-FarmFileDest%>%filter(D.FarmType=="feedlot_cattle_l")
FailedFeeder_s<-DestWarningOnlyCattle%>%filter(DestClass==" feedlot_cattle_s")
FailedFeeder_m<-DestWarningOnlyCattle%>%filter(DestClass==" feedlot_cattle_m")
FailedFeeder_l<-DestWarningOnlyCattle%>%filter(DestClass==" feedlot_cattle_l")
assign_Feeder1 <- function(Failed, Destinations) {
  Failed$Destination <- rep(NA_real_, nrow(Failed))  # Ensure numeric NA

  for (i in 1:nrow(Failed)) {
    src_region <- Failed$Region[i]

    eligible_dests <- Destinations %>% filter(Region == src_region|RegionB=="Feedlot")

    if (nrow(eligible_dests) > 0) {
```

```
      eligible_dests$Destination <- as.numeric(as.character(eligible_dests$Destination
))

      selected <- sample(eligible_dests$Destination, 1)

      Failed$Destination[i] <- selected


    }

  }


  return(Failed)

}

Feedlots_s_merge<-assign_Feeder1(FailedFeeder_s, Feedlot_s)

Feedlots_s_merge<-left_join(Feedlots_s_merge, FarmFileDest, by="Destination")

Feedlots_s_merge<-Feedlots_s_merge%>%rename(Source=FarmID)

Feedlots_s_merge<-Feedlots_s_merge%>%select(DayMov, Source, Destination, S.FarmType, D
.FarmType, EastingS, NorthingS, EastingD, NorthingD, S.State, D.State)

Feedlots_m_merge<-assign_Feeder1(FailedFeeder_m, Feedlot_m)


Feedlots_m_merge<-left_join(Feedlots_m_merge, FarmFileDest, by="Destination")

Feedlots_m_merge<-Feedlots_m_merge%>%rename(Source=FarmID)


Feedlots_m_merge<-Feedlots_m_merge%>%select(DayMov, Source, Destination, S.FarmType, D
.FarmType, EastingS, NorthingS, EastingD, NorthingD, S.State, D.State)

Feedlots_l_merge<-assign_Feeder1(FailedFeeder_l, Feedlot_l)

Feedlots_l_merge<-left_join(Feedlots_l_merge, FarmFileDest, by="Destination")

Feedlots_l_merge<-Feedlots_l_merge%>%rename(Source=FarmID)

Feedlots_l_merge<-Feedlots_l_merge%>%select(DayMov, Source, Destination, S.FarmType, D
.FarmType, EastingS, NorthingS, EastingD, NorthingD, S.State, D.State)

AllFeeders<-bind_rows(Feedlots_s_merge, Feedlots_m_merge, Feedlots_l_merge)


AllFeeders<-AllFeeders%>%mutate(Distance=(sqrt((EastingS-EastingD)^2+(NorthingS-Northi
ngD)^2)))


AllFeedersTab<-AllFeeders%>%group_by(S.FarmType)%>%

  summarise(mean(Distance),

            median(Distance),

            min(Distance),

            max(Distance))


REgionalDistribution<-FarmFileDest %>%
```

```
  group_by(Region, D.FarmType) %>%
  summarise(count = n()) %>%
  arrange(Region)
`summarise()` has grouped output by 'Region'. You can override using the `.groups` arg
ument.
print(REgionalDistribution)
```

There are multiple of each farm type within the regions - the destinations will be assigned within region The non-cattle species will be omitted

```
assign_withinregion <- function(Failed, Destinations) {
  Failed$Destination <- NA  # Add a destination column

  for (i in 1:nrow(Failed)) {
    src_region <- Failed$Region[i]

    # Filter destinations that match region or are in MountainWest
    eligible_dests <- Destinations%>%filter(Region==src_region)

    # Assign destination
    if (nrow(eligible_dests) > 0) {
      selected <- sample(eligible_dests$Destination, 1)
      Failed$Destination[i] <- selected
    }
  }

  return(Failed)
}
table(DestWarningOnlyCattle$DestClass)

FailedCC_l<-DestWarningOnlyCattle%>%filter(DestClass==" cow_calf_l")
FailedCC_s<-DestWarningOnlyCattle%>%filter(DestClass==" cow_calf_s")
FailedDHC_l<-DestWarningOnlyCattle%>%filter(DestClass==" dairy_heifer_calf_l")
FailedDHC_m<-DestWarningOnlyCattle%>%filter(DestClass==" dairy_heifer_calf_m")
FailedDHC_s<-DestWarningOnlyCattle%>%filter(DestClass==" dairy_heifer_calf_s")
FailedDairy_l<-DestWarningOnlyCattle%>%filter(DestClass==" dairy_l")
FailedDairy_s<-DestWarningOnlyCattle%>%filter(DestClass==" dairy_s")
FailedProc_l<-DestWarningOnlyCattle%>%filter(DestClass==" processor_cattle_l")
```

```
FailedProc_s<-DestWarningOnlyCattle%>%filter(DestClass==" processor_cattle")
FailedDealer<-DestWarningOnlyCattle%>%filter(DestClass==" dealer_cattle")
FailedStocker<-DestWarningOnlyCattle%>%filter(DestClass==" stocker_l")
FailedStocker_s<-DestWarningOnlyCattle%>%filter(DestClass==" stocker_s")
```

There were no failed shipments for the following types in this iteration so they are omitted below - Small or Large CC - Small Stocker - Processors of any size - Small Dairies and small dairy heifer calf ranches

```
Dairy_l<-FarmFileDest%>%filter(D.FarmType=="dairy_l")


Dairy_L_merge<-assign_withinregion(FailedDairy_l, Dairy_l)


Dealer<-FarmFileDest%>%filter(D.FarmType=="dealer_cattle")
Dealer_merge<-assign_withinregion(FailedDealer, Dealer)


DHC_L<-FarmFileDest%>%filter(D.FarmType=="dairy_heifer_calf_l")
DHC_M<-FarmFileDest%>%filter(D.FarmType=="dairy_heifer_calf_m")


DHC_L_merge<-assign_withinregion(FailedDHC_l, DHC_L)
DHC_m_merge<-assign_withinregion(FailedDHC_m, DHC_M)


Stocker_L<-FarmFileDest%>%filter(D.FarmType=="stocker_l")
Stocker_L_merge<-assign_withinregion(FailedStocker, Stocker_L)
DestFailsA<-bind_rows(Stocker_L_merge,
                           Dealer_merge,
                           DHC_L_merge,
                           DHC_m_merge,
                           Dairy_L_merge)
DestFails1<-left_join(DestFailsA,FarmFileDest, by="Destination")
DestFails1<-DestFails1%>%rename(Source=FarmID)



DestFails<-bind_rows(DestFails1, Feedlots_l_merge, Feedlots_m_merge, Feedlots_s_merge)
```

Remove the dataframes created that are not needed anymore to prevent mess

```
rm(FailedCC_l, FailedCC_s, FailedDairy_l, FailedDairy_s, FailedDealer, FailedDHC_l, Fa
iledDHC_m, FailedDHC_s, FailedFeeder_l, FailedFeeder_m, FailedFeeder_s, FailedProc_l,
FailedProc_s, FailedStocker, FailedStocker_s);gc()
```

```
rm(Dairy_l, Dairy_L_merge, Dealer, Dealer_merge, Feedlot_l, Feedlot_m, Feedlot_s, Feedlots_s_merge, Feedlots_m_merge, Feedlots_l_merge, Stocker_L, Stocker_L_merge); gc()


rm(DHC_L, DHC_L_merge, DHC_M, DHC_m_merge, DHC_S, DHC_s_merge); gc()
```

The two files are ready to be merged and the markets as nodes added back in

```
DestFails<-DestFails%>%select(DayMov, Source, EastingS, NorthingS, Destination, EastingD, NorthingD, Type, S.FarmType, S.State, D.FarmType, D.State)


OnlyCattle<-OnlyCattle%>%select(DayMov, Source, EastingS, NorthingS, Destination, EastingD, NorthingD, Type, S.FarmType, S.State, D.FarmType, D.State)


AllMovements<-bind_rows(DestFails, OnlyCattle)
```

Sort out the movements from farms to a market that happened in Movement Type 18 (to infect market) and movement 42 (those that passed thru somewhere)

```
MarketFarm<-AllMovements%>%filter(Type=="MovementType18")


MarketThru<-AllMovements%>%filter(Type=="MovementType42") #339,410


Michigan<-MarketThru%>%filter(S.State=="Michigan")


#write.csv(Michigan, "Michigan Movement 42s 60d.2.csv")
```

Select the UP farms in QGIS then reimport – QGIS is a software that can plot the farm locations on the US map built for the farm file.

```
UpperPenn<-read_csv("C:/Users/marykasmith/Desktop/60d Run 2/UP 60.2.csv")
UpperPenn<-UpperPenn%>%filter(DayMov<31)


MarketThru2<-MarketThru%>%filter(!(Source %in% UpperPenn$Source))
```

Did all the farms that made a 42 make a 18?

```
all(MarketThru2$Source %in% MarketFarm$Source) #determines if all Movement 42 Sources are in 18, usually false
[1] FALSE
```

Some sources that had a movement 18 had multiple markets they contacted, sort those out and select the closest one to use as the selected market that movement 42s go to

```
# Find sources with multiple destinations
```

```
multiple_dest_sources <- MarketFarm %>%
  group_by(Source) %>%
  summarize(unique_dest = n_distinct(Destination)) %>%
  filter(unique_dest > 1)
```

Find the distance to the multiple markets contacted in Movement 18

```
MultipleDests<-MarketFarm%>%mutate(Distance=(sqrt((EastingS-EastingD)^2+(NorthingS-NorthingD)^2)))
```

Select the closest one to be the favorite market

```
ClosestMarket<-MultipleDests %>%
  group_by(Source) %>%
  filter(Distance == min(Distance)) %>%
  ungroup()

ClosestMarket<-ClosestMarket%>%select(Source,EastingS,NorthingS,Type,Destination,EastingD,NorthingD,D.State)
ClosestMarket<-ClosestMarket%>%rename(MarketDest=Destination)
```

Remove duplicate shipments to the same market - this is to make the merge to a movement 42 cleaner

```
ClosestMarket <- ClosestMarket %>% distinct(Source, .keep_all = TRUE)
ClosestMarket<-ClosestMarket%>%mutate(D.FarmType="livestock_market_bison_cattle")

MarketThruSource<-MarketThru2%>%select(DayMov,Source,EastingS,NorthingS,S.FarmType, S.State)

AssignMarket<-left_join(MarketThruSource,ClosestMarket,by=c("Source","EastingS","NorthingS")) #needs to equal the number of MarketThru
```

The NA's in the assign market are from farms that did not have a number 18, they will get a random market within their state.

```
NADest<- AssignMarket %>% filter(is.na(MarketDest))
Taggedto18<-AssignMarket%>%filter(!is.na(MarketDest))

Taggedto18<-Taggedto18%>%select(DayMov,Source,EastingS,NorthingS,MarketDest,EastingD,NorthingD,S.FarmType, D.FarmType, S.State, D.State)
Taggedto18<-Taggedto18%>%rename(Destination=MarketDest)
FarmFileSource<-FarmFile%>%select(FarmID, State, Class,Easting,Northing)
```

```
FarmFileSource<-FarmFileSource%>%rename(Source=FarmID)

NeedMarket<-left_join(NADest, FarmFileSource, by="Source")

NeedMarket<-NeedMarket%>%select(DayMov, Source, EastingS, NorthingS, S.FarmType, State
)

NeedMarket<-NeedMarket%>%rename(S.State=State)


NeedMarketSources<-NeedMarket %>% distinct(Source, .keep_all = TRUE)

MarketStates<-FarmFileSource%>%filter(Class=="livestock_market_bison_cattle")

MarketStates<-MarketStates%>%rename(MarketDest=Source,D.State=State)

MatchMarket<-left_join(NeedMarketSources, MarketStates, by=c("S.State"="D.State"))%>%g
roup_by(Source)%>%

  slice_sample%>%ungroup()

Warning: Detected an unexpected many-to-many relationship between `x` and `y`.

MatchMarket<-MatchMarket%>%rename(EastingD=Easting, NorthingD=Northing, D.FarmType=Cla
ss)

MatchMarket<-MatchMarket%>%select(Source, MarketDest, EastingD, NorthingD,D.FarmType)


AssignedStateMarket<-left_join(NeedMarket, MatchMarket, by=c("Source"))

AssignedStateMarket$D.State=AssignedStateMarket$S.State


AssignedWithin1<-AssignedStateMarket%>%filter(!is.na(MarketDest))

AssignedWithin1<-AssignedWithin1%>%rename(Destination=MarketDest)
```

There are states that do not have a market in them in the New England area. They will be assigned to one of the markets that is in that area.

```
Assigned_NA<-AssignedStateMarket%>%filter(is.na(MarketDest))

table(Assigned_NA$S.State)


New Hampshire

          227

Assigned_NA<-Assigned_NA%>%select(-EastingD, -NorthingD, -D.FarmType, -D.State)

assigned_state2 <- Assigned_NA %>%

  rowwise() %>%

  mutate(MarketDest = ifelse(

    any(MarketStates$D.State %in% c("Maine", "New Hampshire", "Vermont","Massachusetts
")),  # Check if states exist

    sample(MarketStates$MarketDest[MarketStates$D.State %in% c("Maine", "New Hampshire
", "Vermont","Massachusetts")], 1),

    NA_real_  # Assign NA if no match found
```

```
  )) %>%
  ungroup()


NewEnglandMarket<-left_join(assigned_state2, MarketStates, by="MarketDest")
NewEnglandMarket<-NewEnglandMarket%>%rename(EastingD=Easting, NorthingD=Northing, D.FarmType=Class,Destination=MarketDest)
wi_markets <- MarketStates %>%
  filter(D.State == "Wisconsin") %>%
  pull(MarketDest)


# Randomly assign one Wisconsin market to each row
UPtoWI <- UpperPenn %>%
  rowwise() %>%
  mutate(MarketDest = sample(wi_markets, 1)) %>%
  ungroup()



UPtoWI<-UPtoWI%>%select(DayMov, Source, EastingS, NorthingS, S.FarmType, S.State, MarketDest)
UPMarket<-left_join(UPtoWI, MarketStates, by="MarketDest")
UPMarket<-UPMarket%>%rename(EastingD=Easting, NorthingD=Northing, D.FarmType=Class,Destination=MarketDest)
```

Add all the lines back together now with markets assigned - these are the movements to market

```
ReassignedMarketState<-bind_rows(NewEnglandMarket, AssignedWithin1, Taggedto18, UPMarket) #again should equal the movement 42 from above
```

The other half of the movement from movement 42 (to the final farm destination) need to be relinked

```
FirstHalf<-ReassignedMarketState%>%rename(Market=Destination,Sourcex=Source) #source x to be the linking piece for ascending order to match the second half
FirstHalf<-FirstHalf%>%select(Sourcex, Market, EastingD,NorthingD)
FirstHalf<-FirstHalf%>%rename(EastingM=EastingD,NorthingM=NorthingD) #M to indicate is the market
FirstHalf <- FirstHalf %>%
  arrange(Sourcex) #this is the to market side


SecondHalf <- MarketThru %>%
  arrange(Source) #this has the final destination
setdiff(FirstHalf$Sourcex, SecondHalf$Source)
```

```
numeric(0)

ToDest<-bind_cols(FirstHalf,SecondHalf) #should equal the market thru

ToDest<-ToDest%>%select(-Source,-Sourcex, -EastingS, -NorthingS, -S.FarmType,-Type)

ToDest<-ToDest%>%rename(Source=Market, EastingS=EastingM, NorthingS=NorthingM)

ToDest<-ToDest%>%mutate(S.FarmType="livestock_market_bison_cattle", Type="FromMarket")
```

Remove the Movement 42s and 18 from the list so they are not duplicated

```
Movementsno42<-AllMovements%>%filter(Type!="MovementType42")

Movementsno42<-Movementsno42%>%filter(Type!="MovementType18")
```

Add all of the movements back together - the original list (Movements no 42), the to market (Reassigned Market) and the second half (ToDest)

```
FinalMovements<-bind_rows(Movementsno42, ReassignedMarketState, ToDest)
```

Write the final movements dataframe to a file as needed for the further analysis.

```
write.csv(FinalMovements,”30DaysNetwork2.csv")
```
