# Supplementary material for: Assessment of cattle traceability using electronic identification and digital movement reporting in simulated United States cattle movement networks
Source: Front Vet Sci. 2026 Oct 27;13:1972037. doi: 10.3389/fvets.2026.1972037 (PMC13747793; doi:10.3389/fvets.2026.1972037)
Supplement: Supplementary file 2 [file Data_Sheet_2.PDF]

Supplement 2: Application of Tagging to Movements

```
library(dplyr)

library(tidyverse)

set.seed(0502)#Used for all networks
```

Load in the movement network (edges) and the farm file

The farm file is needed to match the farmID in the network to the farm type

```
edges<-read_csv("./30DaysNetwork2.csv")
```

```
Rows: 1739723 Columns: 3── Column specification ────────────────────────
────────────────────────────────────────────────────
```

```
farmfile<-read.table("./FarmFile2024MarketsDairies.txt")


farmfiledest<-farmfile%>%rename(D.type=V2)%>%mutate(destination=as.integer(V1
))%>%select(destination,D.type)

ffsource<-farmfiledest%>%rename(source=destination, S.type=D.type)
```

Convert the time to a date. EpiContactTrace requires it as a date. The format does not matter to the rest of the code, changed here just to be consistent.

```
edges$Date <- as.Date("2001-01-01") + edges$time - 1

edges<-edges%>%select(from, to, Date)


edges <- edges %>%
  rename(
    source = from,
    destination = to,
    t=Date
  )


edges$source<-as.integer(edges$source)

edges$destination<-as.integer(edges$destination)


edges<-as.data.frame(edges)
```

The Edges_type is the final step in editing the network to have the information and format needed throughout the remainder of the code

```
edges_dest<-left_join(edges, farmfiledest, by="destination")

edges_type<-left_join(edges_dest, ffsource, by="source")
```

Create a list of source farms and destination farms to assign a tagging and reporting status for phase 1 of traceable network. This represents the farms that have adopted EID and DR technology and apply to all incoming and outgoing movements associated with the farm.

```
sources <- edges_type %>%
  distinct(source, S.type)

dests <- edges_type %>%
  distinct(destination,D.type)
```

The S.Rates and D.Rates are tables built to allow for easier modification of the rates in future scenarios. The tables work as a lookup to connect a farm type with the tagging or reporting rate.

```
S.Rates<-data.frame(

  S.type=c("cow_calf_l","cow_calf_s","dairy_heifer_calf_l","dairy_heifer_calf_m","dairy_heifer_calf_s","dairy_l","dairy_s","dealer_cattle","feedlot_cattle_l","feedlot_cattle_m","feedlot_cattle_s","livestock_market_bison_cattle","processor_cattle","processor_cattle_l","stocker_l","stocker_s"),

  rate=c("tag_cc_l","tag_cc_s","tag_dairy","tag_dairy","tag_dairy","tag_dairy","tag_dairy","tag_dealer","tag_feeder_l","tag_feeder_s","tag_feeder_m","tag_market",0,0,"tag_stocker","tag_stocker"))


D.Rates<-data.frame(

  D.type=c("cow_calf_l","cow_calf_s","dairy_heifer_calf_l","dairy_heifer_calf_m","dairy_heifer_calf_s","dairy_l","dairy_s","dealer_cattle","feedlot_cattle_l","feedlot_cattle_m","feedlot_cattle_s","livestock_market_bison_cattle","processor_cattle","processor_cattle_l","stocker_l","stocker_s"),

  rate=c("report_cc.stocker","report_cc.stocker","report_dairy","report_dairy","report_dairy","report_dairy","report_dairy","report_dealer","report_feeder_l","report_feeder","report_feeder","report_market","report_processor","report_processor","report_cc.stocker","report_cc.stocker"))


tag_cc_s<-0.75
tag_cc_l<-0.75
tag_dairy<-0.95
tag_stocker<-0.02
tag_dealer<-0.02
tag_feeder_s<-0.01
tag_feeder_m<-0.01
tag_feeder_l<-0.02
tag_market<-0.1
```

```
report_cc.stocker<-0.9
report_dairy<-0.9
report_feeder<-0.9
report_feeder_l<-0.9
report_dealer<-0.9
report_market<-0.9
report_processor<-0.9
```

Farms are assigned as a tagging farm (1) or not (0)

```
SourcesTag <- sources %>%
  left_join(S.Rates, by = "S.type") %>%
  # Convert rate variable name to actual numeric value
  mutate(rate = sapply(rate, get),
         tagfarm  = rbinom(n(), size = 1, prob = rate))

SourcesTag<-SourcesTag%>%select(source,tagfarm)
```

Farms are assigned as a reporting farm (1) or not (0)

```
DestReport <- dests %>%
  left_join(D.Rates, by = "D.type") %>%
  # Convert rate variable name to actual numeric value
  mutate(rate = sapply(rate, get),
         repfarm  = rbinom(n(), size = 1, prob = rate))

DestReport<-DestReport%>%select(destination,repfarm)
```

Assign that status to all movements to and from the farms

```
Taggedmoves <- edges_type %>%
  right_join(SourcesTag, by = "source") %>%
  right_join(DestReport, by = "destination") %>%
  mutate(
    tagged = as.integer(tagfarm == 1),
    reported = as.integer(repfarm == 1)
  )
table(Taggedmoves$tagged)
```

```

     0      1 
907870 831853 
```

This is completes Phase 1 of tagging.

Begin Phase 2 of Tagging

Begin by calculating the outgoing tag probability using the incoming tagged movements divided by the total incoming movements

```
ingoingrates <- Taggedmoves %>%
  group_by(destination) %>%
  summarise(
    D.type = first(D.type),
    total = n(),
    taggedin = sum(tagged == 1),
    inrate = taggedin / total,
    .groups = "drop"
  )
average_inrate <- ingoingrates %>%
  group_by(D.type) %>%
  summarise(
    mean_inrate = mean(inrate, na.rm = TRUE),
    .groups = "drop"
  )


#Rename to apply to the outgoing movements of that farm type or individual fa
rm


average_inrate<-average_inrate%>%rename(S.type=D.type)

indiv_inrate<-ingoingrates%>%rename(source=destination)
```

Apply this in a binomial to the outgoing movements that are now leaving that farm as a source

```
Taggedmoves2 <- Taggedmoves %>%
  left_join(
    indiv_inrate %>% select(source, inrate),
    by = "source"
  ) %>%
  left_join(
    average_inrate %>% select(S.type, mean_inrate),
    by = "S.type"
  ) %>%
  mutate(
    tagged2 = case_when(
      tagged == 1 ~ 1,
      tagged == 0 & tagfarm == 0 & !is.na(inrate) ~
        rbinom(n(), 1, inrate),
      tagged == 0 & tagfarm == 0 & is.na(inrate) ~
        rbinom(n(), 1, mean_inrate),
      TRUE ~ tagged
    )
  )
table(Taggedmoves2$tagged2) #total non tagged and tagged
```

```
      0       1

 378745 1360978
```

Finally, filter the movements that were tagged and reported (traceable) and write the final csv that contains the network for later FOI tracing efforts.

```
Traceable<-Taggedmoves2 %>%
    mutate(Traced = if_else(tagged2 == 1 & reported == 1, 1, 0)) %>%
    filter(Traced == 1)


Dailycount<-Traceable%>%count(t)
summary(Dailycount$n)
```

```
   Min. 1st Qu.  Median    Mean 3rd Qu.    Max. 
  36779   40444   41759   41136   42235   42892 
```

```
write.csv(Traceable,"CC7590_final_Network.csv")
```

Begin Phase 3 of tagging

Calculate for all farms the percent of received contacts that were tagged

```
pct_table <- Taggedmoves2 %>%

  group_by(destination) %>% #the farm being investigated
  summarise(
    total = n(),
    tagged1 = sum(tagged2 == 1),
    outgolikely =  tagged1 / total
  )
pct_table<-pct_table%>%rename(source=destination)
```

The outgolikely is the # tagged/# total movements received

Apply this in a binomial to the outgoing movements that are now leaving that farm as a source

```
Taggedmoves3 <- Taggedmoves2 %>%

  left_join(
    pct_table %>% select(source, outgolikely),
    by = "source"
  ) %>%
  mutate(
    tagged3 = case_when(
      tagged2 == 1 ~ 1,
      tagged2 == 0 & tagfarm == 0 & !is.na(outgolikely) ~
        rbinom(n(), 1, outgolikely),
      TRUE ~ tagged2
    )
  )
```

This completes phase 3 of tagging application.

Finally, filter the movements that were tagged and reported (traceable) and write the final csv that contains the network for later FOI tracing efforts.

```
Traceable<-Taggedmoves3 %>%

    mutate(Traced = if_else(tagged3 == 1 & reported == 1, 1, 0)) %>%

    filter(Traced == 1)


Dailycount<-Traceable%>%count(t)

summary(Dailycount$n) #average daily movements for curiosity sake

   Min. 1st Qu.  Median    Mean 3rd Qu.    Max.

  36164   41783   43123   42432   43857   44970

write.csv(Traceable,"CC7590_final_Network.csv")
```
