# Supplementary material for: Assessment of cattle traceability using electronic identification and digital movement reporting in simulated United States cattle movement networks
Source: Front Vet Sci. 2026 Oct 27;13:1972037. doi: 10.3389/fvets.2026.1972037 (PMC13747793; doi:10.3389/fvets.2026.1972037)
Supplement: Supplementary file 3 [file Data_Sheet_3.PDF]

Cow Calf Tracing Example

Definition:

FOI: the farmID for a farm of interest that is “infected” and being traced Market: A livestock market where cattle were sold Direct movement: A movement from one farm to another (no markets) Edges file: The dataframe with the movement record Farmfile: The reference that contains the farm type information that matches the IDs in the edges files

General Approach

1. Find all movements from markets that have the FOI as the destination
2. Search the contacts that directly moved to the markets in the same week that the foi contacted the market(s)
3. Search the entire 28 days without markets These are the farm to farm movements
4. Add the number of traces found in each search back together as the total number for each FOI.

```
library(dplyr)
library(tidyverse)
library(EpiContactTrace)
library(readr)
library(lubridate)
library(purrr)
```

Load in the network, farmfile, and farms that serve as the FOIs

```
edges<-read_csv("./CC7590_final_Network.csv")

ccsample<-read_csv("./CowCalfSampleN2D30_100.csv") #These are the FOIs

edges<-edges%>%select(source, destination, t, S.type, D.type)

edges$source<-as.integer(edges$source)

edges$destination<-as.integer(edges$destination)

edges_type<-as.data.frame(edges)
```

STEP 1: Identify all incoming market movements to the FOIs

```
df_market_destination <- edges_type %>%
  filter(destination %in% ccsample$node,
         S.type == "livestock_market_bison_cattle") %>%
  select(destination, source, D.type, S.type, t) %>%
  mutate(unique_id = row_number())%>%
  mutate(market_id=source)


# Peek at the results

head(df_market_destination)
```

destination <int>	source <int>	D.type <chr>	S.type <chr>	t <date>	unique_id <int>	market_id <int>
45638	1243503	cow_calf_l	livestock_market_bison_cattle	2001-01-20	1	1243503
275006	1243784	cow_calf_s	livestock_market_bison_cattle	2001-01-04	2	1243784
32848	1243791	cow_calf_l	livestock_market_bison_cattle	2001-01-15	3	1243791
32848	1243787	cow_calf_l	livestock_market_bison_cattle	2001-01-03	4	1243787
344095	1243268	cow_calf_s	livestock_market_bison_cattle	2001-01-06	5	1243268
42043	1243845	cow_calf_l	livestock_market_bison_cattle	2001-01-29	6	1243845

6 rows

BEGIN STEP 2: Market Contacts

```
# initialize an empty tibble

filtered_edges <- tibble::tibble(FOI = integer(), edges = character(), unique
_id = integer()) #uniqueIDs are needed in the event of multiple market moveme
nts per FOI


for (i in seq_len(nrow(df_market_destination))) {
  unique_id <- df_market_destination$unique_id[i]
  market    <- df_market_destination$source[i]
  dest      <- df_market_destination$destination[i]

  result <- edges_type %>%
    mutate(cut = case_when(
      destination == market ~ 0,
      D.type == "livestock_market_bison_cattle" ~ 1,
      TRUE ~ 0
    )) %>%
    filter(cut == 0) %>%
    mutate(unique_id = unique_id)

  # Build object name
  obj_name <- paste0("edges_", dest, "_", unique_id)

  # Save dataframe in global environment
  assign(obj_name, result, envir = .GlobalEnv)

  # Append new row to the accumulator
  filtered_edges <- dplyr::bind_rows(
    filtered_edges,
    tibble::tibble(FOI = dest, df_edges = obj_name, unique_id = unique_id)
  )
}
```

Merge the results back to link with the FOI

```
matchedFOItime<-right_join(filtered_edges,df_market_destination, by="unique_i
d")%>%select(FOI, df_edges, unique_id, source, t)

print(matchedFOItime)
```

FOI <int>	df_edges <chr>	unique_id <int>	source <int>	t <date>
45638	edges_45638_1	1	1243503	2001-01-20
275006	edges_275006_2	2	1243784	2001-01-04
32848	edges_32848_3	3	1243791	2001-01-15
32848	edges_32848_4	4	1243787	2001-01-03
344095	edges_344095_5	5	1243268	2001-01-06
42043	edges_42043_6	6	1243845	2001-01-29
45350	edges_45350_7	7	1243853	2001-01-06
42043	edges_42043_8	8	1243841	2001-01-06
16924	edges_16924_9	9	1243703	2001-01-12
32848	edges_32848_10	10	1243004	2001-01-05

1-10 of 22 rows

STEP 2: Search the market contacts

```
pwalk(
  list(
    matchedFOItime$df_edges,
    matchedFOItime$FOI,
    matchedFOItime$unique_id,
    matchedFOItime$source,
    matchedFOItime$t
  ),
  function(df_edges_name, FOI, uid, source_mrk, current_t) {

    # get the edges dataframe
    df_edges_obj <- get(df_edges_name, envir = .GlobalEnv)

    # find incoming contacts to the source farm
    Farm_FOI <- df_edges_obj %>%
      filter(
        destination == source_mrk,
        t <= current_t,
        t >= (current_t - 7)
      ) %>%
      select(destination, source, D.type, S.type, t)

    # build output dataframe
    contact_df <- data.frame(
```

```
      source = as.character(Farm_FOI$source),
      destination = as.character(Farm_FOI$destination),
      t= Farm_FOI$t,
      stringsAsFactors = FALSE
    )

    # create object name
    obj_name <- paste0("contact_df_mrk_", FOI, "_", uid)

    # save to global environment
    assign(obj_name, contact_df, envir = .GlobalEnv)
  }
)
```

BEGIN STEP 3: Direct Farm to Farm Contacts

Remove all markets - to have the direct farm to farm shipments only - 28 days duration

```
edges_filtered<-edges_type%>%filter(D.type!="livestock_market_bison_cattle")
#removes movements with markets as destinations

edges_filtered<-edges_filtered%>%filter(S.type!="livestock_market_bison_cattl
e") #removes movements with markets as sources

pwalk(
  list(ccsample$node),
  function(node) {
    # run Trace
    Farm_FOI <- Trace(
      edges_filtered,
      node,
      tEnd = "2001-01-30",
      days = 28
    )

    # build output dataframe from ingoingContacts
    contact_df <- data.frame(
      source      = Farm_FOI@ingoingContacts@source,
      destination = Farm_FOI@ingoingContacts@destination,
      time        = Farm_FOI@ingoingContacts@t,
      stringsAsFactors = FALSE
    )

    # build the object name
    obj_name <- paste0("contact_df_direct_", node)

    # assign it to global env
    assign(obj_name, contact_df, envir = .GlobalEnv)
  }
)
```

The results of this tracing event will stay in the global environment to be referenced later.

At this point there are a lot of intermediate files that can be cleaned up and need to be to have the final counts added together

```
rm(list = ls(pattern = "edges", envir = .GlobalEnv), envir = .GlobalEnv)

# run garbage collection
gc()
```

STEP 4: Adding the number of contacts count all together

```
walk(ccsample$node, function(n) {
  # find objects with this node in their name
  obj_names <- ls(pattern = n, envir = .GlobalEnv)

  # filter objects that are dataframes and have the expected columns
  obj_names <- keep(obj_names, ~ {
    obj <- get(.x, envir = .GlobalEnv)
    is.data.frame(obj) && all(c("source", "destination") %in% names(obj))
  })

  # pull them into a named list
  dfs <- map(set_names(obj_names), ~ get(.x, envir = .GlobalEnv))

  # bind them together
  final_df <- bind_rows(dfs, .id = "source_df")

  # assign into global env with name like "final_48992"
  assign(paste0("final_", n), final_df, envir = .GlobalEnv)
})
```

Find the unique ID count. This is the number of unique farms that the FOI contacted. The n_rows is the total number of movements that were made.

```
node_summary <- map_dfr(ccsample$node, function(n) {
  df_name <- paste0("final_", n)

  # Check if the dataframe exists
  if (!exists(df_name, envir = .GlobalEnv)) {
    return(tibble(node = n, n_rows = NA_integer_, unique_farmids = NA_integer
_))
  }

  df <- get(df_name, envir = .GlobalEnv)

  # Validate columns
  if (!all(c("source", "destination") %in% names(df))) {
    return(tibble(node = n, n_rows = NA_integer_, unique_farmids = NA_integer
_))
  }

  # Handle empty dataframes gracefully
  if (nrow(df) == 0) {
    return(tibble(node = n, n_rows = 0, unique_farmids = 0))
  }

  # Count rows
  n_rows <- nrow(df)
```

```
  # Count unique farm IDs across both columns, excluding the node itself
  unique_count <- df %>%
    select(source, destination) %>%
    unlist(use.names = FALSE) %>%
    unique() %>%
    setdiff(n) %>%   # exclude the node itself if it appears
    length()

  tibble(node = n, n_rows = n_rows, unique_farmids = unique_count)
})

print(node_summary)
```

node <dbl>	n_rows <dbl>	unique_farmids <dbl>
626317	1	1
33338	101	99
44509	127	72
252577	1	1
194401	0	0
42043	214	123
7986	1	1
351536	104	56
48992	0	0
15991	75	40

...
1-10 of 100 rows

Save the node summary for later to reference in the tagged/reported and traceable networks

```
node_summary<-node_summary%>%mutate(Scenario="CC7590")
write.csv(node_summary,"100CC_cc7590_final.csv")
```
