# Supplementary material for: Assessment of cattle traceability using electronic identification and digital movement reporting in simulated United States cattle movement networks
Source: Front Vet Sci. 2026 Oct 27;13:1972037. doi: 10.3389/fvets.2026.1972037 (PMC13747793; doi:10.3389/fvets.2026.1972037)
Supplement: Supplementary file 4 [file Data_Sheet_5.PDF]

Supplementary Figures

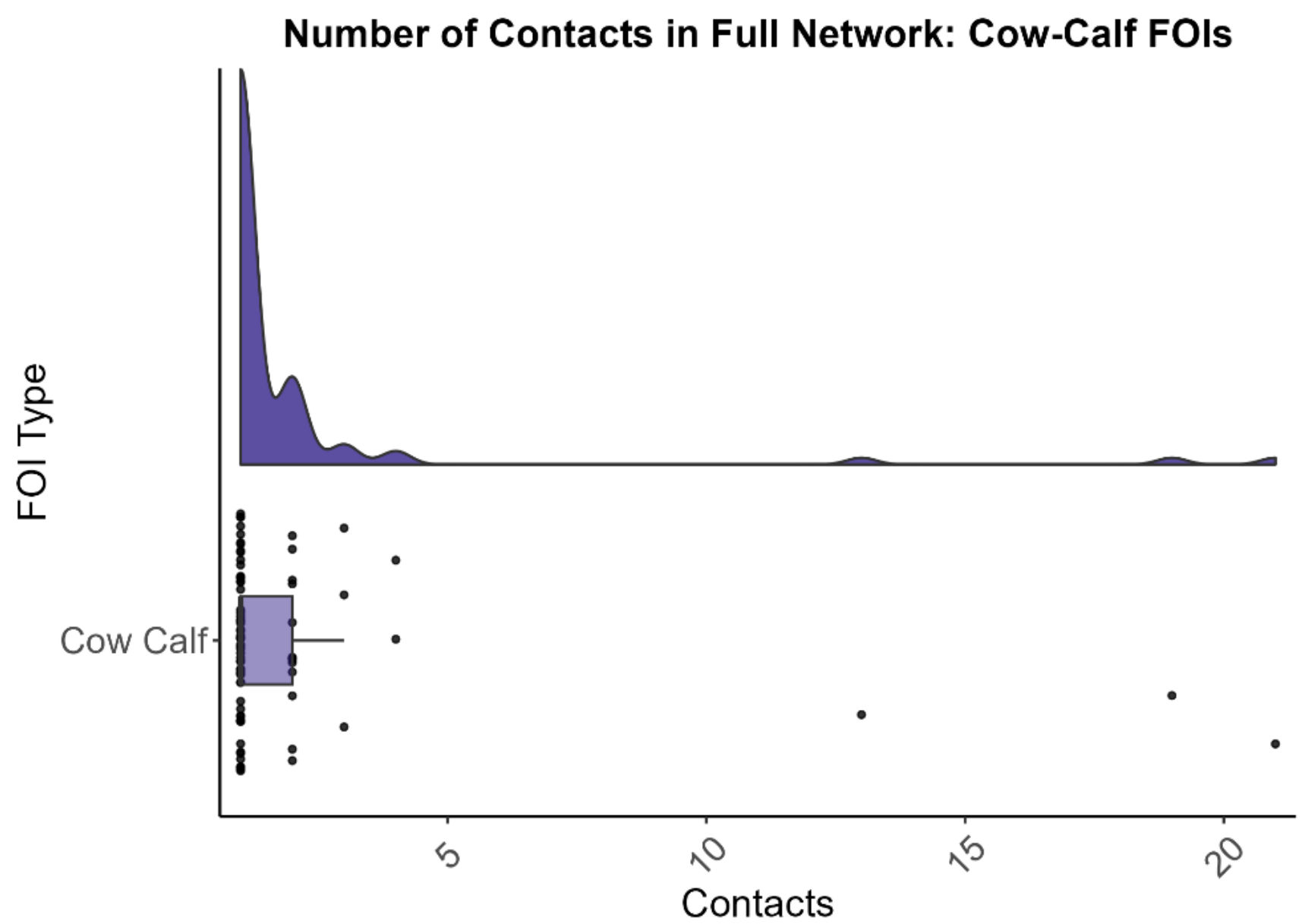

Figure 1: Distribution of contacts in the full network for cow-calf farm FOIs. The distribution has been truncated to those that have less than 25 contacts in this figure(n=80). The range of all cow-calf FOI contacts in the full network extended to 560 contacts.

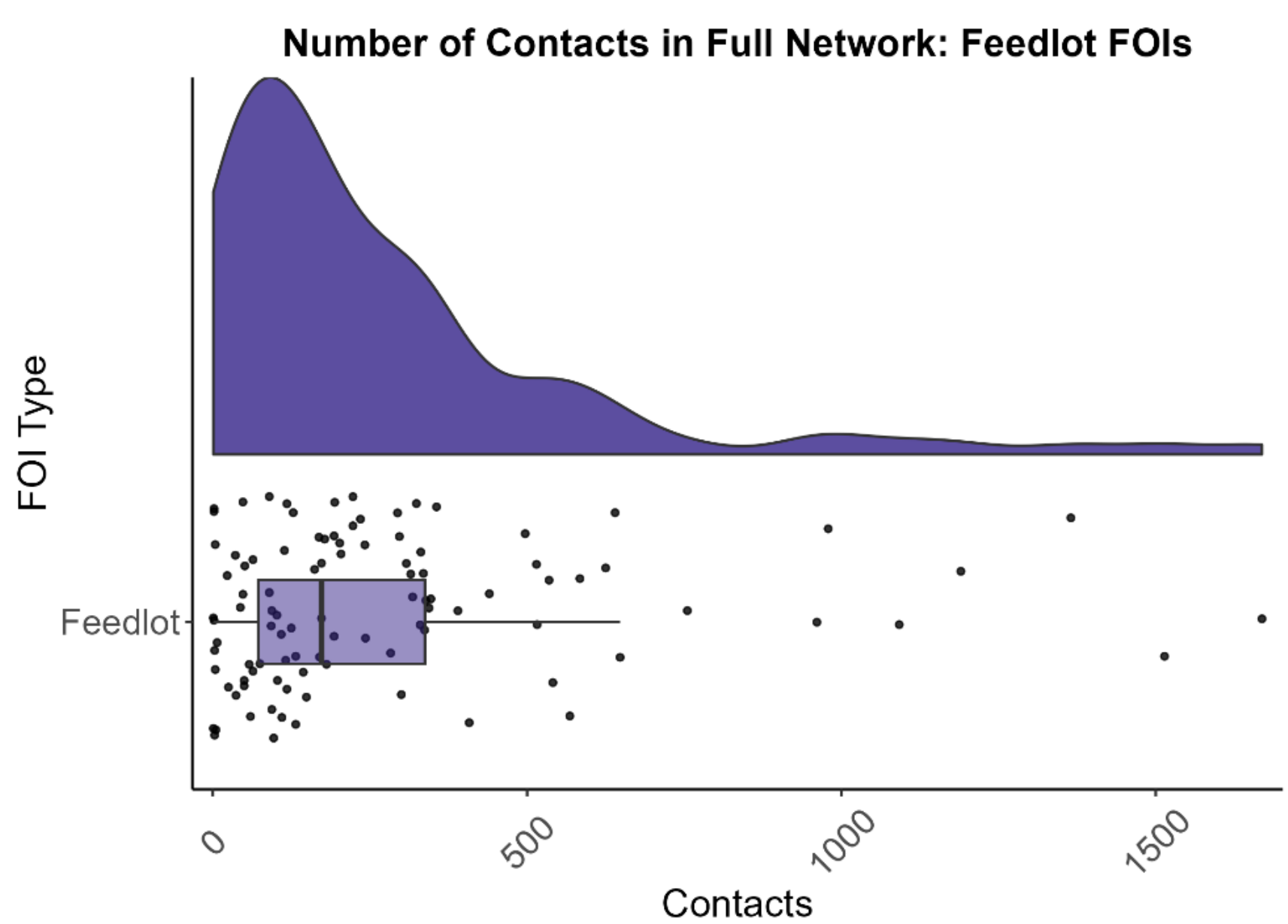

Figure 2: Distribution of contacts in the full network for feedlot FOIs. No truncation applied (n=100).

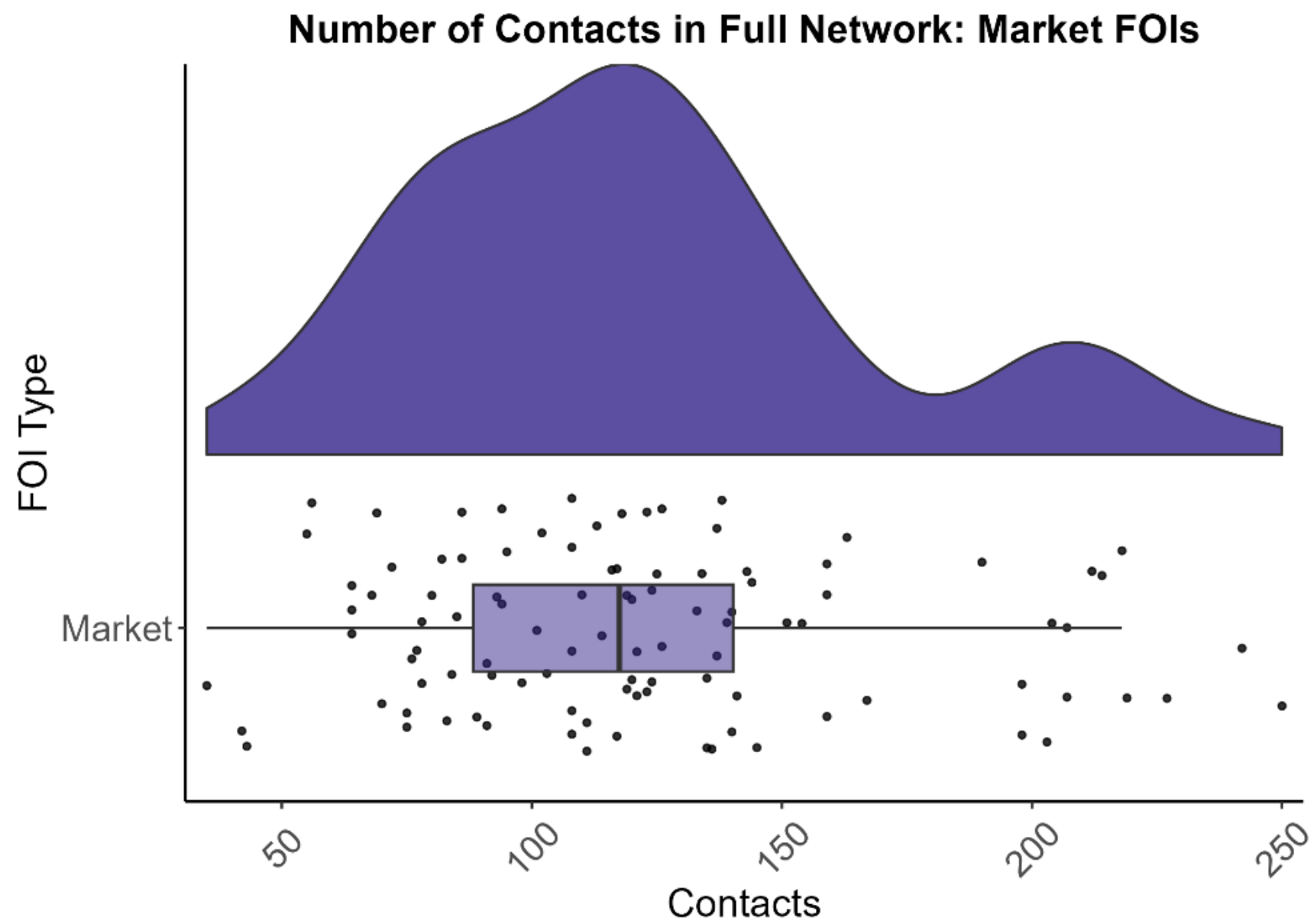

Figure 3: Distribution of contacts in the full network for market FOIs. No truncation applied (n=100).

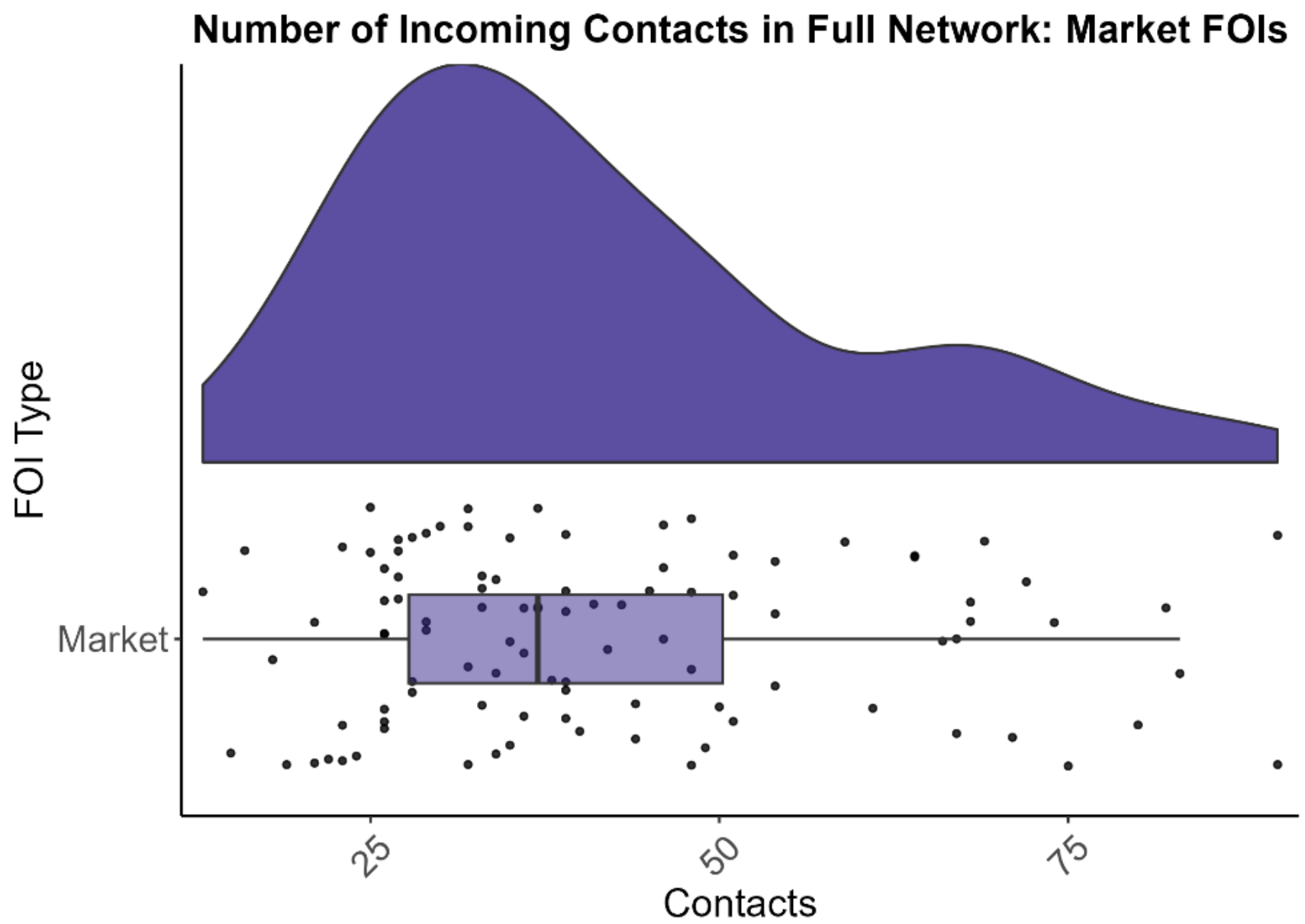

Figure 4: Distribution of incoming contacts in the full network for market FOIs. No truncation applied (n=100).

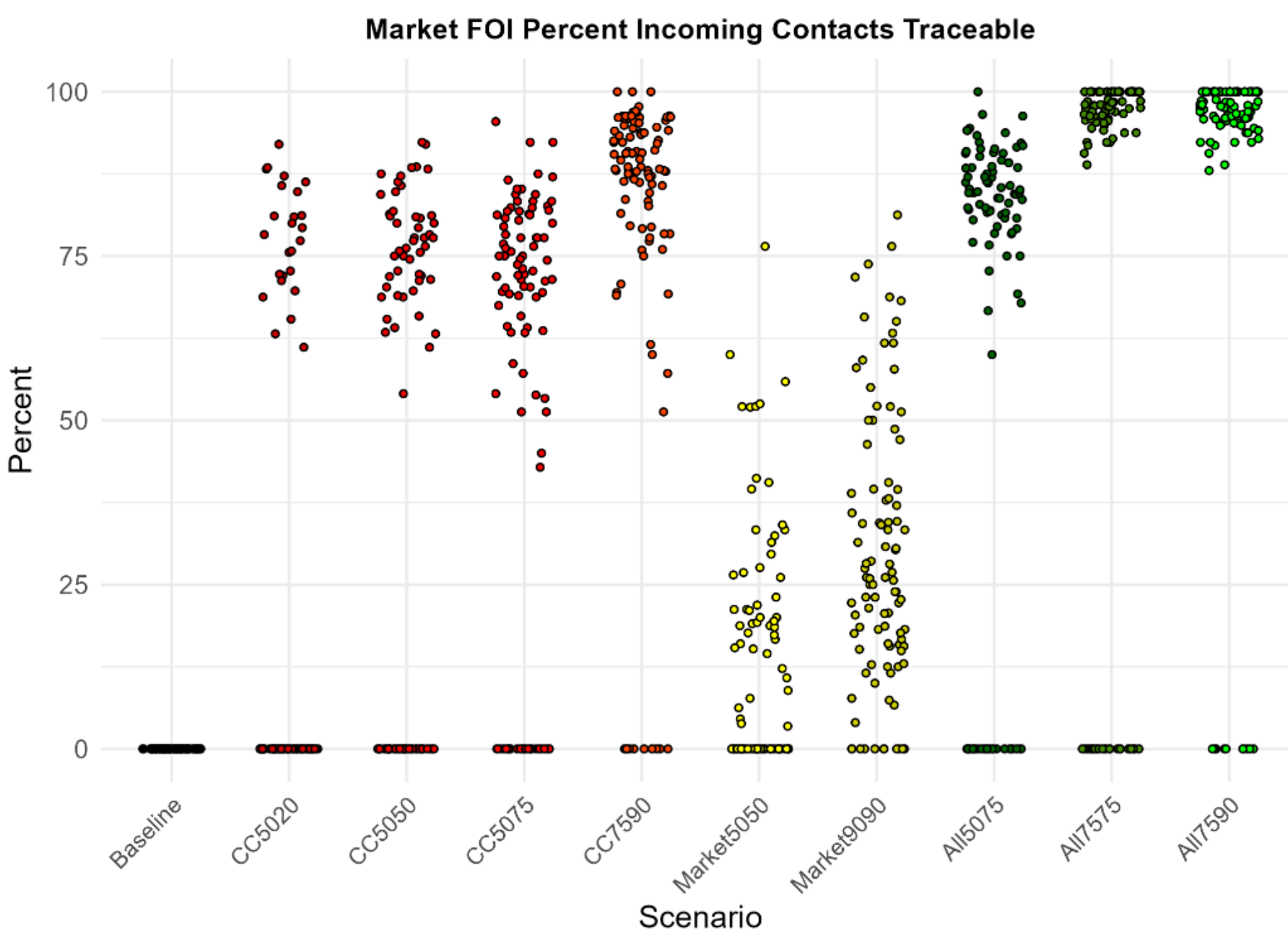

Figure 5: The percent of incoming contacts from market FOIs that were traceable (colored circles) in each scenario.

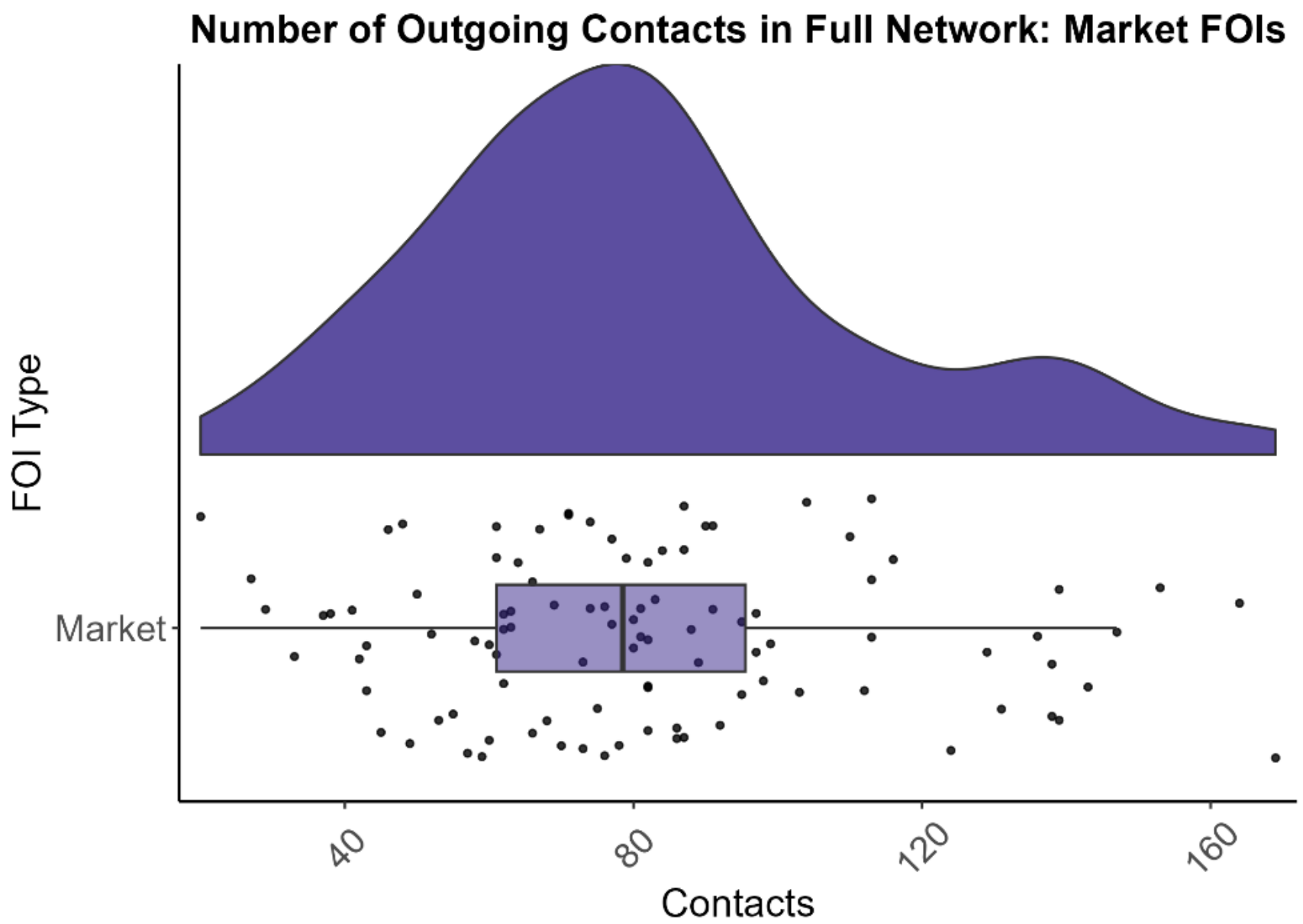

Figure 6: Distribution of outgoing contacts in the full network for market FOIs. No truncation applied (n=100).

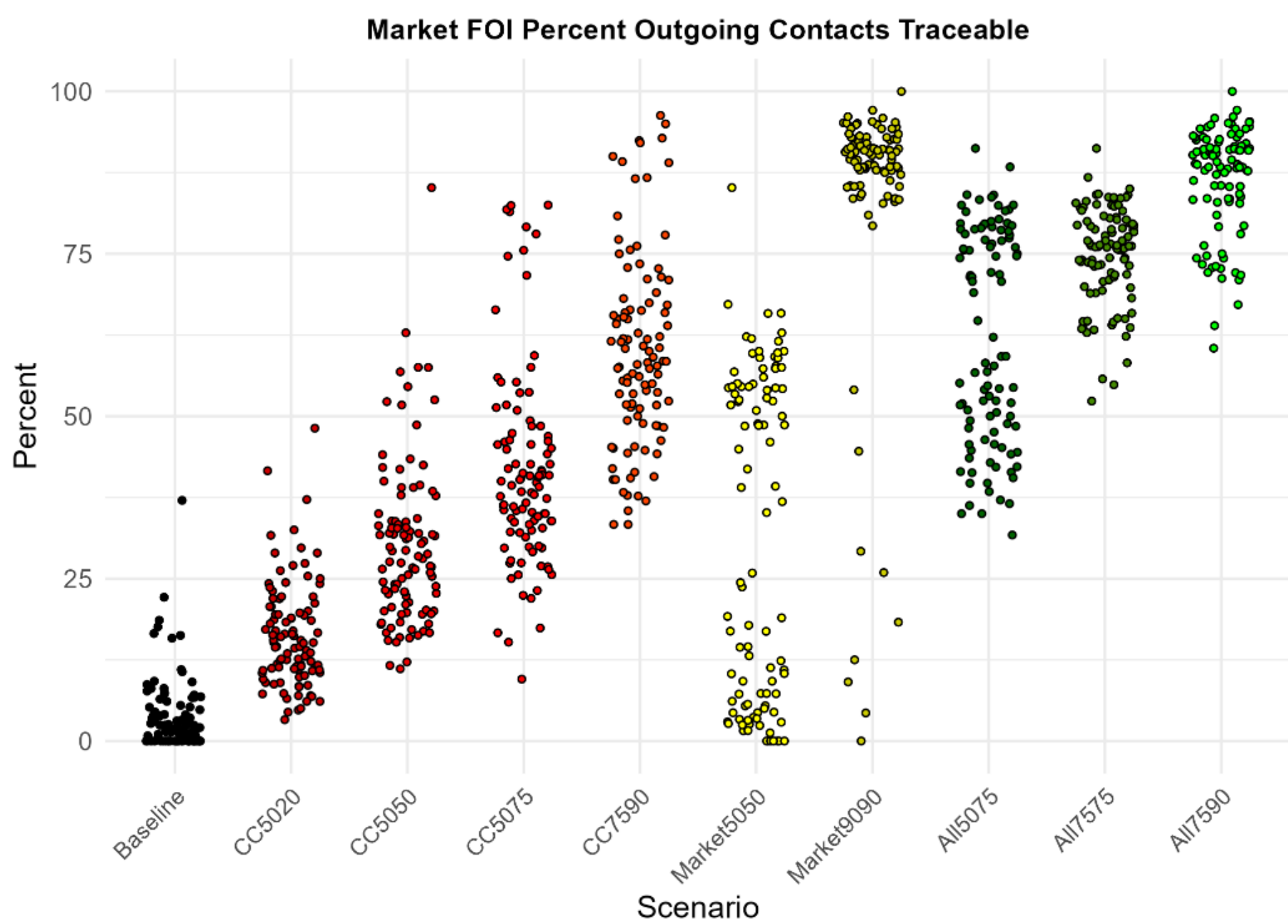

Figure 7: The percent of outgoing contacts from market FOIs that were traceable (colored circles) in each scenario.
